# The impact of face-to-face social exclusion on university students’ interpersonal cooperation behavior: a hyperscanning study based on fNIRS

**DOI:** 10.3389/fnins.2026.1807205

**Published:** 2026-05-25

**Authors:** Huiling Wang, Lin Li, Changjiang Liu

**Affiliations:** 1 School of Physical Education, Xi’an Jiaotong University, Xi’an, China; 2 Key Laboratory of Adolescent Health Evaluation and Exercise Intervention, Ministry of Education, East China Normal University, Shanghai, China; 3 School of Physical Education and Health, East China Normal University, Shanghai, China

**Keywords:** fNIRS, interpersonal neural synchronization (INS), interpersonal cooperation, Prisoner’s Dilemma task, social exclusion

## Abstract

**Background:**

Most studies on social exclusion adopt virtual paradigms focusing on unilateral responses, while neglecting real-world face-to-face interaction and its neural basis. Functional near-infrared spectroscopy (fNIRS) hyperscanning allows recording of interpersonal neural synchronization (INS) during dyadic interaction, providing a novel approach for investigating interpersonal cooperation.

**Methods:**

This study recruited 24 dyads of college students randomly assigned to social exclusion and inclusion groups. Using fNIRS hyperscanning combined with a face-to-face rejection paradigm and the Prisoner’s Dilemma task, we examined subjective experience, behavior, and INS.

**Results:**

(1) The exclusion group reported lower intimacy, trust, belonging need and state self-esteem than the inclusion group. (2) Only defection decision reaction time was faster in the exclusion group, with no group differences in overall cooperation and defection rates, indicating exclusion primarily accelerates defection. (3) INS showed channel-specific differences: the exclusion group had weaker right orbitofrontal INS during cooperation and stronger left dorsolateral prefrontal INS during defection. (4) Cooperation reaction time negatively correlated with trust, while defection efficiency positively correlated with left frontopolar INS. (5) State self-esteem partially mediated the link between social exclusion and defection reaction time.

**Conclusion:**

From an integrated psychological–behavioral–neural perspective, this study confirms that face-to-face social exclusion accelerates defection decisions by impairing subjective interpersonal experience, altering prefrontal INS, and through the mediating effect of subjective feelings. These findings provide empirical evidence for understanding the mechanisms underlying campus social exclusion.

## 1 Introduction

Interpersonal cooperation, as a core form of social interaction, runs through the entire course of human social development. Its essence is to achieve a win-win outcome for individuals and collectives through collaborative actions (Gonzalez, 1991). However, cooperation is not always stable. Social exclusion phenomena such as isolation, defection, rejection and discrimination commonly exist in daily life, which damage harmonious interpersonal relationships and become critical obstacles to cooperation. Social exclusion refers to a process in which individuals are rejected or excluded by social groups or others, resulting in the impairment of their needs for belonging and social connection (Du and Xia, 2008). It manifests in diverse forms, including refusal, ostracism and neglect (Molden et al., 2009), and different exclusion paradigms may trigger distinct motivational responses (Yang et al., 2019). Compared with other experimental paradigms, the face-to-face social exclusion paradigm features immediate emotional feedback and direct interactive impact, exerting stronger negative effects, threatening individuals’ core psychological needs, and further interfering with interpersonal cooperation (Shao and Zhang, 2021).

The General Aggression Model proposes that individuals who experience face-to-face rejection are more likely to exhibit withdrawal or aggressive behaviors toward the rejectors (Williams, 2008; Williams, 2009). Meanwhile, social exclusion profoundly reshapes individuals’ subjective social experiences. According to Williams’ Need to Belong Theory, social exclusion directly threatens four fundamental psychological needs: belongingness, meaningful existence, sense of control, and state self-esteem, thereby increasing aggressive tendencies and reducing prosocial behaviors, especially cooperation. Accumulating evidence indicates that social exclusion significantly reduces interpersonal trust, intimacy and cooperative willingness. Defensive emotions induced by exclusion may further promote defection behaviors to cope with social threats and inhibit cooperation (Williams and Nida, 2022; Wirth et al., 2017; Zhu et al., 2025). Specifically, trust serves as the foundation of interpersonal cooperation; social exclusion undermines positive social expectations and reduces trust and cooperative intentions. Additionally, exclusion weakens interpersonal closeness and enlarges social distance, which further restricts cooperative motivation. Subjective cooperation, as a direct reflection of internal cooperative tendencies, complements objective behavioral indicators (Walasek et al., 2019). These subjective experiences are core psychological variables linking social exclusion to interpersonal decision-making and are indispensable for clarifying the underlying mechanisms of exclusion (Cheng et al., 2011). Therefore, this study systematically measured intimacy, trust, subjective cooperation, need to belong and state self-esteem to comprehensively depict the psychological outcomes of social exclusion, and proposed the first hypothesis:

*Hypothesis* 1 (H1): Social exclusion significantly reduces multiple dimensions of subjective experience, including intimacy, trust, subjective cooperation, need to belong, and state self-esteem.

Further explore the neural mechanisms underlying the association between social exclusion and cooperation, this study adopted functional near-infrared spectroscopy (fNIRS) hyperscanning. This technique enables real-time monitoring of brain activities during dyadic interactions, with high ecological validity for simulating natural social scenarios (Montague et al., 2002; Cui et al., 2012). Interpersonal neural synchronization (INS), the core indicator of fNIRS hyperscanning, has been recognized as a critical neural marker for evaluating interpersonal interaction quality (Mueller et al., 2021). Previous studies have confirmed that enhanced prefrontal INS is closely linked to cooperative behaviors (Zhang et al., 2019; Zhang et al., 2020) and the Prisoner’s Dilemma task is widely applied to assess real-time cooperative and defective decision-making (Peng et al., 2019). Existing neuroimaging studies have demonstrated that social exclusion primarily activates prefrontal-related networks, including the default mode network, salience network and executive control network, which participate in emotional and cognitive processing of social pain (Onoda et al., 2010). Nevertheless, most prior studies focus on individual brain activation, while neglecting dyadic INS alterations under social exclusion from a hyperscanning perspective. Given the pivotal role of the prefrontal cortex in social decision-making, combined with the advantages of fNIRS hyperscanning, the second hypothesis was proposed:

*Hypothesis* 2 (H2): Social exclusion significantly alters prefrontal interpersonal neural synchronization during cooperative and defective decision-making, presenting distinct channel-specific patterns.

Although the Need to Belong Theory provides a fundamental framework for understanding the psychological consequences of social exclusion, two major research gaps remain. First, most studies focus on the direct linkage between social exclusion and isolated behaviors, failing to systematically clarify the mediating role of subjective experience or the integrated associations among subjective perception, behavioral performance and inter-brain neural synchronization. Second, existing neural research predominantly adopts single-brain imaging techniques, which only capture individual neural activation and ignore the inherent dyadic coordination of interpersonal interaction, making it difficult to reveal the inter-brain mechanisms of cooperation and defection under exclusion. Accordingly, the current study combined a high-ecological-validity face-to-face rejection paradigm with fNIRS hyperscanning, constructing an integrated framework of “social exclusion – subjective experience – dyadic neural synchronization – interpersonal decision-making.” Beyond single-path and single-brain research, this study focused on the mediating effect of subjective experience and identified channel-specific INS changes during cooperation and defection, so as to supplement and expand the theoretical boundary of the Need to Belong Theory. On this basis, Hypothesis 3 (H3) was proposed:

*Hypothesis* 3 (H3): Subjective experiences (e.g., trust, belongingness and intimacy) mediate the effects of social exclusion on interpersonal cooperation; meanwhile, social exclusion reshapes prefrontal INS, which is significantly correlated with behavioral indicators.

College students, in a critical period of personality development and social relationship establishment, are a high-risk group for social exclusion. Frequent campus isolation and marginalization impair their cooperative ability, threaten physical and mental health and social participation, and may even induce antisocial behaviors with potential risks to social stability. Exploring the impacts of face-to-face social exclusion on cooperative and defective behaviors among college students, as well as its psychological and neural mechanisms, contributes to understanding campus social conflicts and providing theoretical support for improving students’ social adaptability.

In summary, based on a randomized controlled design and fNIRS hyperscanning technology, this study explored the effects of face-to-face social exclusion on interpersonal cooperation among college students, and systematically interpreted the underlying psychological-neural mechanisms from subjective, behavioral and inter-brain perspectives. The findings offer novel insights for research on social exclusion and interpersonal interaction, and provide empirical references for campus mental health intervention.

## 2 Participants and methods

### 2.1 Participants

In the present study, a total of 24 dyads of college students were recruited, with a mean age of 19.04 ± 1.25 years. The sample size was consistent with the conventional sample range of existing fNIRS hyperscanning studies focusing on interpersonal interaction and cooperative neural mechanisms (Acconito et al., 2026; Ushijima et al., 2026). To strictly control potential confounding variables, purify the core causal relationship between social exclusion and interpersonal cooperation, and ensure baseline homogeneity between groups, standardized and rigorous inclusion criteria were adopted, and all sampling restrictions were clearly set for experimental control purposes.

First, all dyadic interactions were limited to same-sex pairs. Previous social cognitive studies have demonstrated that sex differences significantly affect interpersonal distance, social expectation, cooperative preference, and individual sensitivity to social exclusion. Cross-sex interaction may introduce additional confounding factors, such as sex stereotypes and biased social anticipation, which could alter individuals’ subjective exclusion experience and cooperative decision-making patterns. Since the core focus of this study was to investigate the pure effect of social exclusion on behavioral performance and inter-brain synchronization (INS), rather than sex differences, only same-sex dyads were recruited to eliminate sex-related interference.

Second, only only-child participants were included. Family rearing background profoundly shapes individual social patterns, conflict coping styles, and interpersonal tolerance. Compared with only children, non-only children generally accumulate richer experience in daily interpersonal negotiation, compromise, and cooperation through sibling interaction, and exhibit higher tolerance toward negative social feedback and social exclusion. In contrast, only children have relatively narrower daily social experience and are more sensitive to external social evaluation and social exclusion. To unify the baseline of interpersonal traits and eliminate confounding effects caused by family socialization differences, only only-child participants were recruited in this study.

Third, all participants were required to have no long-term professional sports training experience. Existing evidence indicates that long-term sports experience can significantly improve individuals’ cooperative tendency and behavioral performance, as well as enhance prefrontal INS during cooperative interaction. Such individual differences may interfere with the relationship between social exclusion and subsequent cooperative behaviors. Therefore, this strict screening criterion was applied to eliminate sports-related confounding effects and ensure consistent neural and behavioral baselines across groups.

Furthermore, personality traits and sex distribution were strictly balanced in this study. A total of 12 dyads were assigned to the exclusion group and another 12 dyads to the inclusion group, with equal distributions of introversion/extroversion personality and sex to further control individual differences. All participants were right-handed, with normal or corrected-to-normal vision and no color blindness. They reported no physical illness, neurological disorders, psychiatric problems, or recent major negative life events. None of the participants had taken part in similar social interaction experiments before. After the experiment, a post-experimental suspicion check was conducted to exclude participants who perceived the false social feedback, ensuring the validity of the final data.

All participants provided written informed consent before the experiment and received corresponding remuneration after completing the tasks. This study was approved by the Human Experimental Ethics Committee (Approval No. HR500–2023) and strictly followed the ethical principles outlined in the latest version of the Declaration of Helsinki.

### 2.2 Materials and apparatus

1. Questionnaires and scales.

This study adopted a series of standardized scales for participant screening, baseline matching, manipulation checks, and pre- and post-experimental state assessment. Confounding variables including personality, emotion, physical activity, and chronic social experiences were strictly controlled to ensure intergroup homogeneity and experimental internal validity. The specific function of each scale is described as follows:

1. Demographic Information Form: Collects basic information such as sex, age, and psychiatric history for preliminary participant screening.
2. International Physical Activity Questionnaire-Short Form (IPAQ-SF; Booth, 1996): Assesses daily physical activity levels to achieve baseline matching and control the confounding effects of exercise differences on interpersonal interaction and emotional states (Qiu et al., 2022).
3. Cooperative and Competitive Personality Scale (CCPS; Xie et al., 2006) and Eysenck Personality Questionnaire-Adult (EPQ-A; Eysenck et al., 1991): Used for personality trait screening and classification to exclude individuals with extreme personalities and balance personality differences across groups.
4. Intimacy Scale, Trust Scale, Subjective Cooperativeness Scale: Administered before and after the experiment to measure subjective social experiences and verify the effectiveness of social exclusion manipulation (Li et al., 2020).
5. Self-Rating Anxiety Scale (SAS; Zung, 1971), Beck Depression Inventory-II (BDI-II; Beck et al., 1997), Interaction Anxiousness Scale (IAS; Leary, 1983): Applied to screen emotional problems and exclude participants with obvious anxiety, depression, and social anxiety tendencies.
6. College Students’ Social Exclusion Questionnaire (Wu et al., 2013): Evaluates chronic social exclusion experiences and eliminates individuals with high chronic exclusion to reduce baseline individual differences.
7. Profile of Mood States-Short Form (POMS-SF): Measures negative emotional states and screens participants with stable mood (McNair, 1984; Zhu, 1995).
8. Need to Belong Scale (Leary et al., 1995; NTB) and State Self-Esteem Scale (Heatherton and Polivy, 1991; SSES): Repeatedly administered before and after the experiment to examine changes in belonging motivation and state self-esteem induced by social exclusion.

Detailed information regarding item composition, scoring rules, reverse scoring, classification criteria, exclusion thresholds, and psychometric properties of all scales is presented in “8. Questionnaires and Scales” of Supplementary material.

1. Interpersonal Cooperation Paradigm—Prisoner’s Dilemma Task.

**Figure 1:**
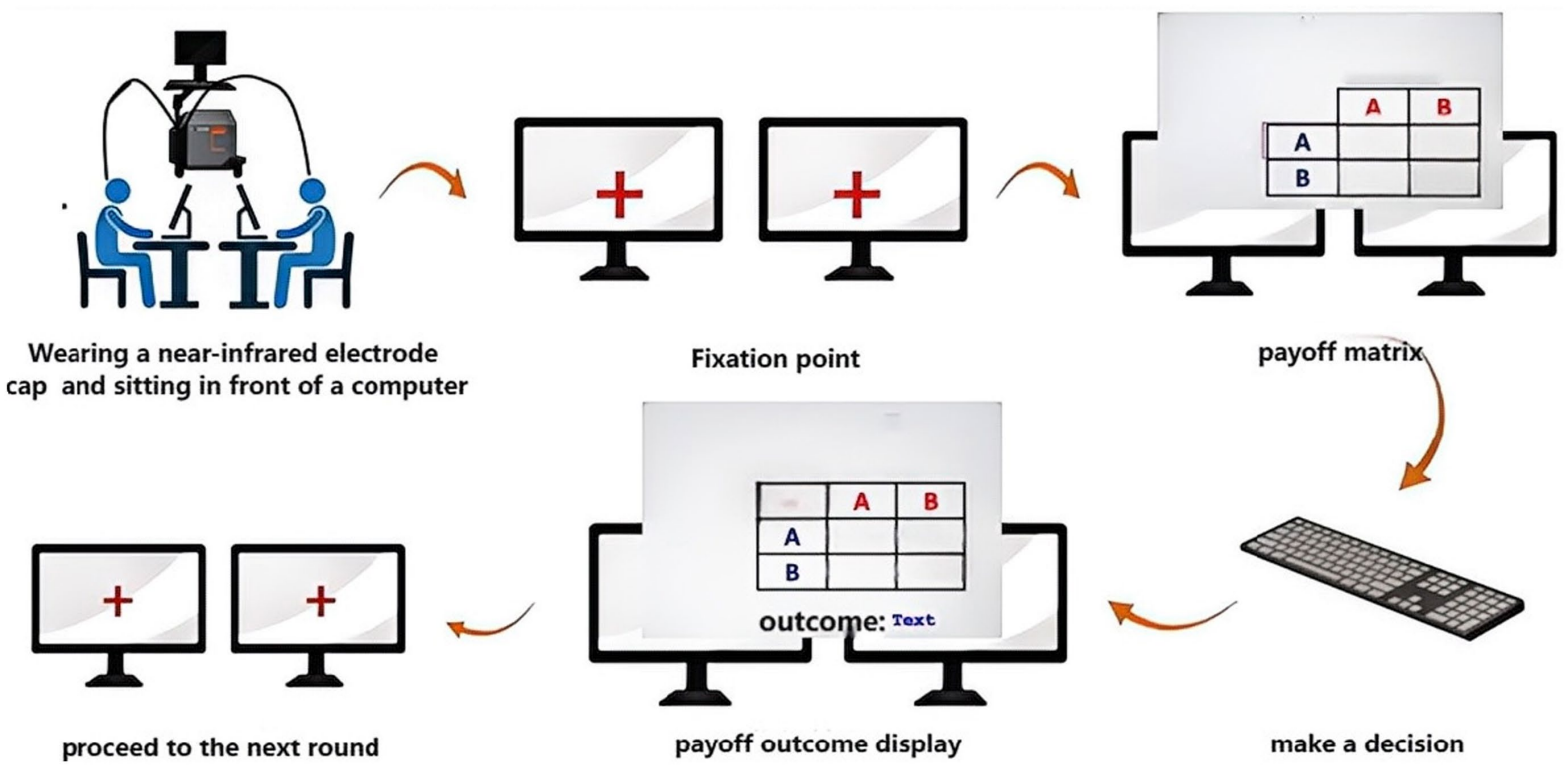
Flowchart of the Prisoner’s Dilemma task.

The experiment adopted a monetary game task paradigm adapted from the classic Prisoner’s Dilemma game and programmed via E-Prime 2.0 (Rilling et al., 2002). In this paradigm, two participants completed multiple rounds of interactive decision-making. In each trial, both players independently chose to either cooperate or defect, with corresponding monetary payoffs assigned according to their joint choices. If both participants chose cooperation, each obtained payoff R; if both chose defection, each obtained payoff P; if one player cooperated and the other defected, the defector obtained payoff F, while the cooperator obtained payoff H. The payoff structure followed two standard rules: (1) F > R > P > H; (2) 2R > H + F. Accordingly, individual expected payoffs were higher under defection conditions (F and P) than under cooperation conditions (R and H), while mutual cooperation yielded a higher total collective payoff for the dyad compared with mutual defection. This setting created a typical social dilemma, in which participants aimed to maximize their personal gains. To avoid financial losses caused by the partner’s defection, both participants needed to strategically prevent being exploited by their counterpart. Participants were informed in advance that their final accumulated task payoffs would be converted into real monetary rewards at a conversion ratio of 50%, which was designed to enhance their sense of immersion and authenticity during interpersonal interaction. See Figure 1.

1. Materials for the Social-Exclusion Manipulation.

Three same sex participants were arranged to enter the laboratory simultaneously in this experiment. All roles were preset in advance rather than assigned randomly according to arrival order. Specifically, two individuals were formal real participants, whose serial numbers were randomly assigned by the experimenter to ensure group balance. The third participant was a pre-arranged experimental confederate who strictly followed the experimental procedure throughout the task, effectively eliminating the confounding influence of role assignment bias on interpersonal interaction and social exclusion manipulation. All three individuals sat face-to-face with full visual access to one another in a real offline interactive environment. They first engaged in a 10-min free conversation to enhance mutual familiarity and establish a genuine temporary small-group interaction atmosphere.

After the familiarity phase, each participant was required to select their preferred partner from the other two members for the subsequent two-person game task. All selection outcomes and social feedback were presented through a WeChat-like simulated text interface. Standardized false feedback was uniformly delivered by the experimenter in the background. Social manipulation was achieved solely through unified textual information, which eliminated confounding non-verbal cues such as vocal tone, facial expressions, and body postures, thereby ensuring the consistency and controllability of experimental manipulation across the two groups.

**Figure 2:**
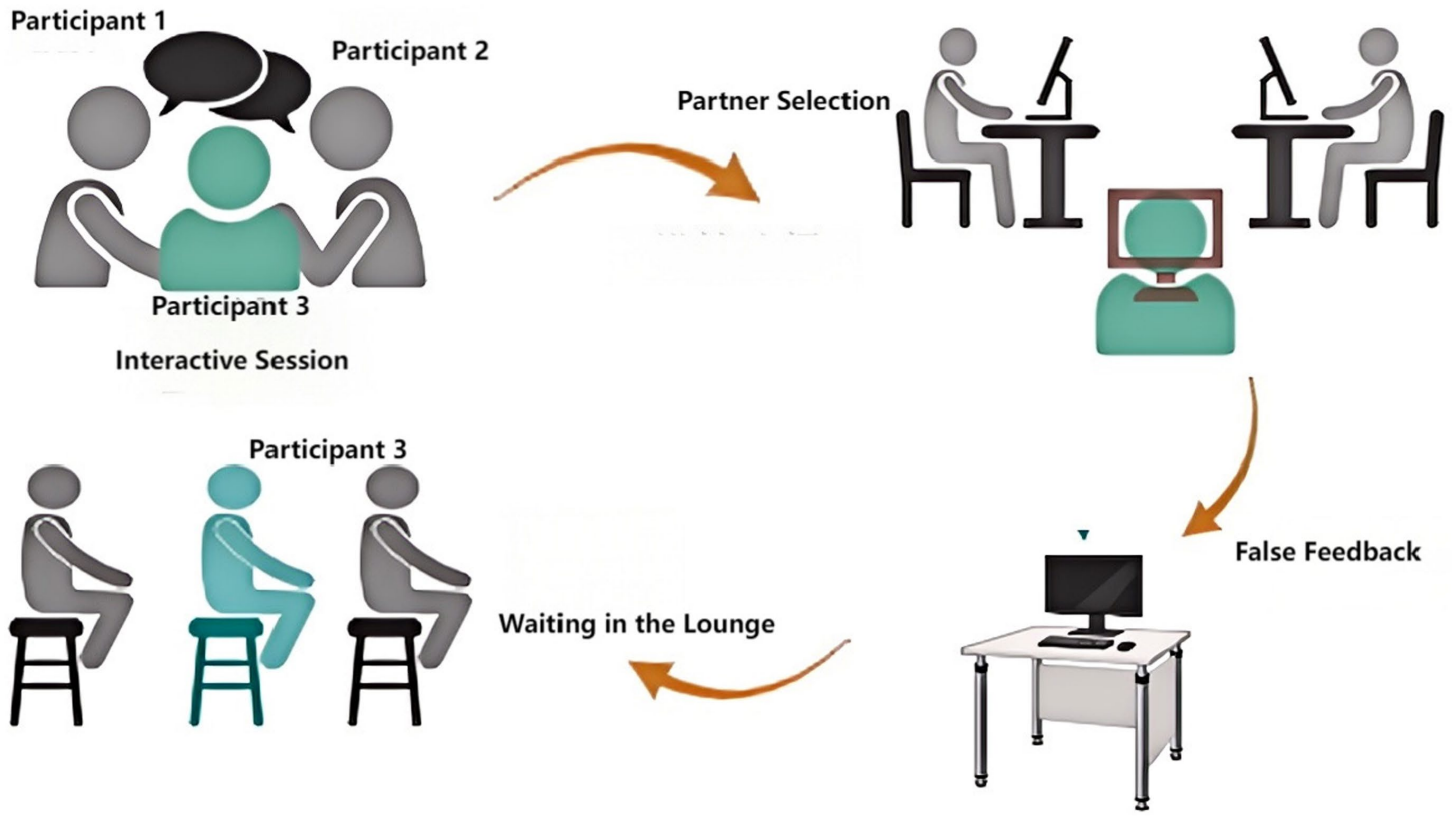
Flowchart of the social-exclusion manipulation.

In terms of specific manipulation procedures, real participants in the exclusion group received two consecutive rejection feedbacks from their partners to induce persistent feelings of social exclusion. In contrast, participants in the inclusion group obtained positive team acceptance feedback to maintain a normal and friendly social experience, forming a valid control condition. The manipulation procedure of the present study is consistent with the classical minimal group paradigm. The social interaction situation was constructed based on classic operations including temporary group formation, peer preference selection, and interpersonal social evaluation. Widely adopted in studies on social categorization, interpersonal acceptance, and social exclusion, this paradigm can stably and effectively induce distinct social experiences, providing solid theoretical and paradigmatic support for the design of the social exclusion task in this study. See Figure 2 for details.

### 2.3 Experimental procedure

**Figure 3:**
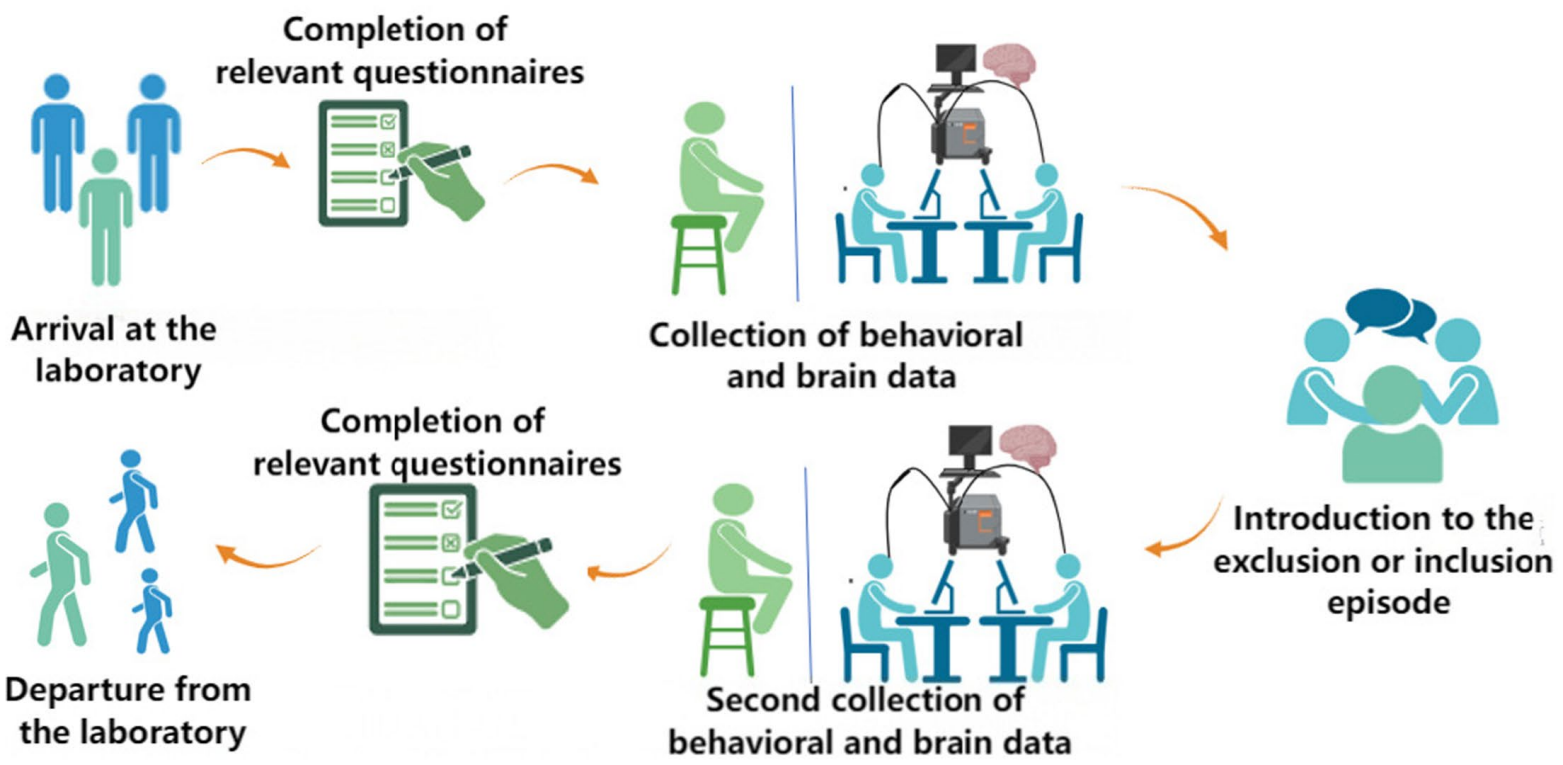
Flowchart of the experimental procedure.

See Figure 3. Upon arrival, the three participants signed the informed-consent form and completed a demographic questionnaire (including age, sex, athletic experience, etc.) and the full set of scales. Participant 3 (the confederate) waited in the lounge for 10 min, while Participants 1 and 2 wore fNIRS optodes to record brain activity, then completed the Prisoner’s Dilemma task and filled in the emotion questionnaires.

Before the task, Participants 1 and 2 sat face-to-face (their view blocked by a screen). After the experimenter confirmed the fNIRS signal was normal, the task started: 30-s baseline rest → 2-s red fixation cross → key press choice within 5–10 s (Participant 1: 1 = cooperate, 2 = defect; Participant 2: 4 = cooperate, 5 = defect) → 3-s outcome presentation. A total of 30 trials were conducted (≈ 7 min). Participants were informed that the final payment = base fee + a percentage of the points earned.

After the task, Participants 1 and 2 removed the optodes, and Participant 3 re-entered the laboratory. The three chatted for 10 min to enhance familiarity, then selected a preferred partner via WeChat. The experimenter sent false feedback (exclusion group = rejected, inclusion group = accepted), and participants completed manipulation-check and mood questionnaires in separate lounges while waiting.

Subsequently, Participants 1 and 2 returned to the laboratory, re-wore the fNIRS optodes, and completed the second Prisoner’s Dilemma task and post-manipulation questionnaires. After all tasks, the true purpose of the study was explained, apologies and compensation were offered, and participants were asked not to disclose the deception. They were debriefed and dismissed one by one, and the session ended.

### 2.4 Data acquisition

1. Behavioral data acquisition.

The Prisoner’s Dilemma task was programmed with E-Prime 3.0 (Psychology Software Tools, Inc.). Recorded variables included mean cooperation rate, mean defection rate, reaction time (RT) for cooperative choices, RT for defection choices, cooperation efficiency, and defection efficiency.

1. fNIRS data acquisition.

**Figure 4:**
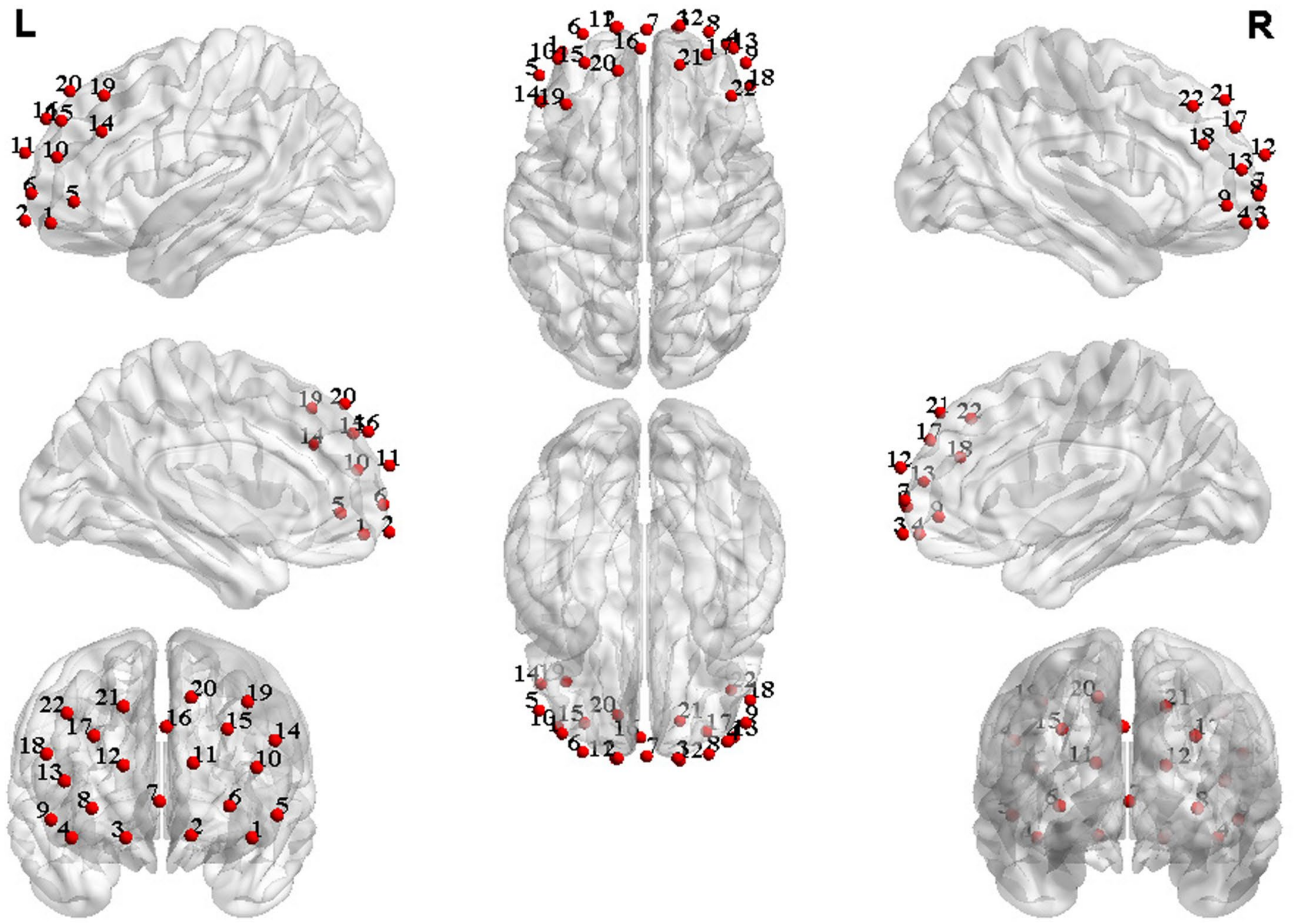
Spatial location map of the prefrontal cortex.

Cerebral data were collected with a Hitachi ETG-7100 system, which monitors changes in oxygenated (HbO) and deoxygenated (HbR) hemoglobin concentrations within the prefrontal cortex. The device employs two wavelengths (695 nm and 830 nm) at a sampling rate of 10 Hz. An optode patch placed across the forehead contained 8 emitters and 7 detectors (inter-optode distance = 3 cm), yielding 22 measurement channels, see Figure 4. Following the international 10–20 system, the central detector was positioned at Fpz; the midline optode row was aligned along the Nasion–Inion line. Channel locations were referenced to the Jichi University anatomical template in Supplementary Table 1.

### 2.5 Statistical analysis

1. Behavioral-data analysis.

All statistical analyses were performed using SPSS 26.0 and Hayes’s PROCESS procedures (Andrew F. Hayes, Ph. D.). All analyses were conducted at the dyadic level (*N* = 24 dyads). For individual questionnaire indicators, the average score of each dyad was calculated and converted into dyad-level variables. Behavioral indicators generated during dyadic interpersonal interactions were directly treated as dyad-level variables. In the present study, the independent variable was social exclusion manipulation (group: exclusion group = 1, inclusion group = 0). The mediating variables included subjective experience (i.e., intimacy, trust, sense of belonging, state self-esteem, and subjective cooperation) and INS. The dependent variables consisted of behavioral indicators, including average cooperation rate, average defection rate, average cooperation reaction time, average defection reaction time, cooperation efficiency, and defection efficiency. Pearson correlation analysis was used to examine pairwise correlations among independent, mediating, and dependent variables, so as to clarify the basic association patterns for subsequent mediation modeling.

The formulas for behavioral indicators were defined as follows:

Average cooperation rate (%) = number of mutual cooperation trials / total trials × 100;

Average defection rate (%) = number of mutual defection trials / total trials × 100;

Average cooperation reaction time (ms) = total reaction time of mutual cooperation decisions / corresponding decision times;

Average defection reaction time (ms) = total reaction time of mutual defection decisions / corresponding decision times;

Cooperation efficiency = average cooperation rate / average cooperation reaction time (faster responses indicate higher cooperation efficiency);

Defection efficiency = average defection rate / average defection reaction time (faster responses indicate higher defection efficiency).

A 2 (group: exclusion vs. inclusion) × 2 (time: pre-test vs. post-test) repeated-measures analysis of variance was adopted to test the main and interactive effects of group and time on all variables. Furthermore, Bootstrap mediation analysis (Model 4) was performed to examine the mediating roles of subjective experience and INS in the relationship between social exclusion and interpersonal cooperative behavior. Direct effects, indirect effects, and 95% confidence intervals were reported accordingly.

1. fNIRS data analysis

All data preprocessing procedures were performed based on MATLAB (2014a) scripts. Given that oxyhemoglobin (HbO) is more sensitive to task-related stimuli (Hoshi, 2003), only HbO signals were analyzed in the present study, which is consistent with previous fNIRS hyperscanning studies (Hu et al., 2017).

First, principal component analysis (PCA) spatial filtering was applied to preprocess the raw fNIRS data. To eliminate signal interference caused by environmental fluctuations, the HbO concentration signals recorded during the 30-s resting state before the Prisoner’s Dilemma task were adopted as the experimental baseline. PCA was further used to filter out noise originating from head movements and systemic physiological fluctuations in HbO time series (Zhang et al., 2016).

Second, wavelet transform coherence (WTC) analysis was employed to calculate the temporal correlation of HbO concentration change time series between each dyad. WTC is a reliable method for measuring the cross-correlation of two time series across varying time and frequency domains. The WTC analysis was implemented using the MATLAB function package for cross-wavelet and wavelet coherence analysis. For each channel of every dyad under each task condition, two HbO time series were extracted and organized for WTC calculation, generating time-frequency coherence maps after signal transformation.

**Figure 5:**
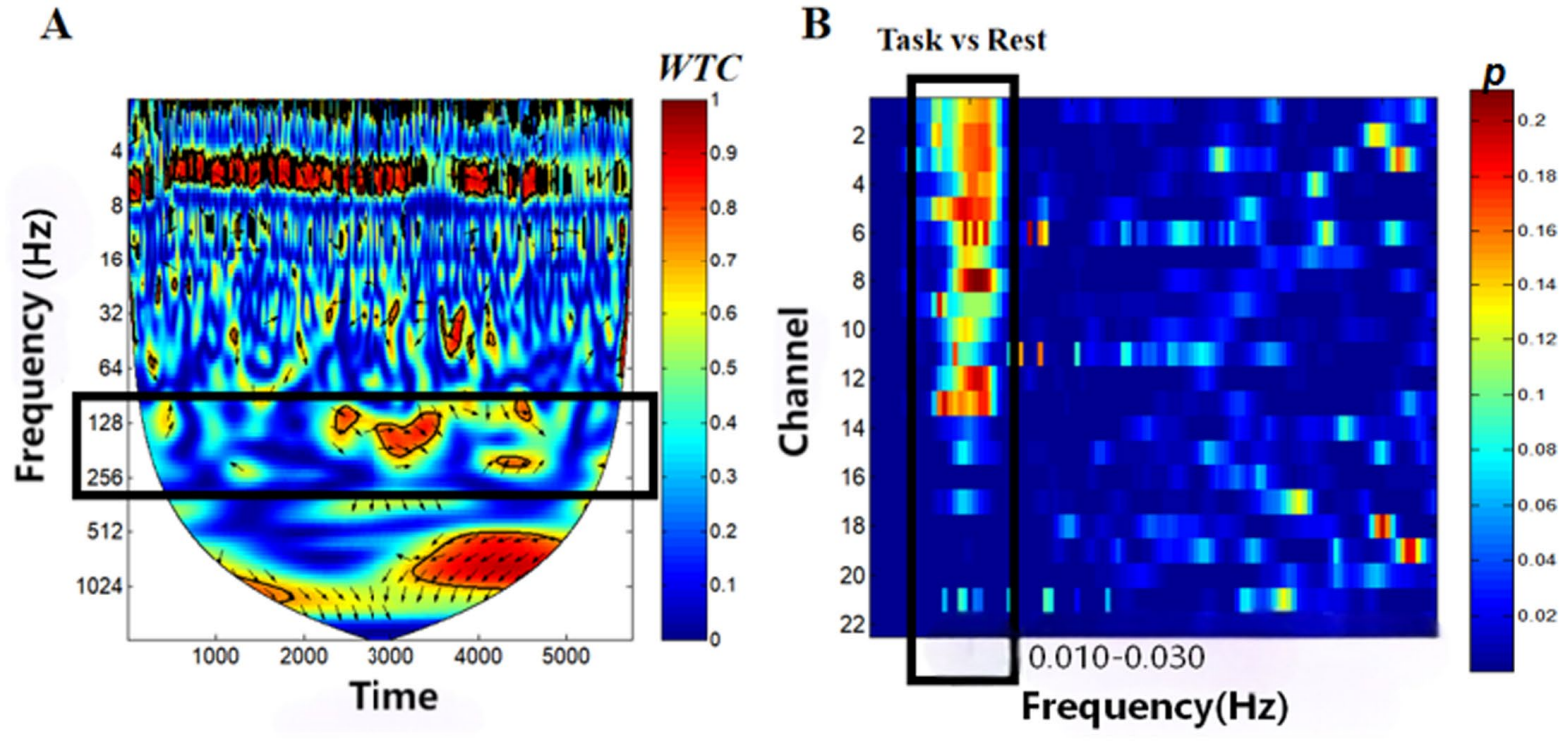
Time-frequency coherence maps of Hbo signals based on wavelet transform coherence (WTC; data from a randomly selected dyad). **(A)** WTC spectrum of HbO signals in specific channels of the social exclusion group. The color gradient ranges from dark blue (synchronization value = 0) to deep red (synchronization value = 1), representing the degree of signal synchronization, and the black rectangles mark regions with significant INS. **(B)** WTC spectrum of the exclusion group within the frequency band of 0.001 Hz–0.1 Hz. The INS in the frequency band of 0.010 Hz–0.030 Hz during the task period was significantly higher than that during the resting period, and this frequency band fully covered the duration of the decision-making phase.

During data analysis, the frequency band with significant task-stage coherence rather than resting-stage coherence was selected, ranging from 0.01 Hz to 0.03 Hz (corresponding to a time window of 10.0 s to 30.0 s), as illustrated in Figure 5A. This time-frequency range matched the duration of each game round (approximately 20 s). Moreover, this frequency band effectively eliminated high-frequency noise in NIRS signals and was consistent with the band selection criteria of previous fNIRS motor studies (Holper et al., 2012).

To further verify the task-specific frequency band, a broader frequency range of 0.001–0.1 Hz (corresponding to a time window of 1 ~ 100 s) was selected for supplementary analysis according to prior research (Cui et al., 2012). The mean INS values within this band were calculated to identify dominant coherent intervals. The results confirmed the strongest coherence within the 10.0 s ~ 30.0 s time window (Figure 5B), which was consistent with the initially defined region of interest.

Consistent with existing hyperscanning evidence, interpersonal interaction elicits higher inter-brain coherence than the resting state (Jiang et al., 2015). Therefore, task-related coherence was defined as the difference value between task-stage coherence and resting-state coherence, followed by Fisher z-transformation for standardized statistical analysis. Subsequently, xjview and BrainNet Viewer toolboxes were used for brain data visualization (Xia et al., 2013).

Finally, the preprocessed INS data were extracted and subjected to a 2 (Group: exclusion vs. inclusion) × 2 (Time: pre-test vs. post-test) repeated-measures analysis of variance to examine the effects of group and time on each indicator. Subsequent analytical procedures were consistent with those adopted for the behavioral data.

## 3 Results

### 3.1 Demographic and baseline characteristics

To equate individual differences between the exclusion and inclusion groups, participants were pre-screened with the battery listed in Supplementary Table 4. Independent-samples t-tests on age, extraversion/introversion (EPQ-E), IPAQ activity level, cooperative and competitive tendencies, SAS anxiety, BDI depression, interaction anxiousness, chronic social-exclusion score, POMS negative affect, and pre-experiment intimacy, trust, and perceived cooperativeness revealed no significant between-group differences (*p* > 0.05), confirming baseline homogeneity.

### 3.2 Subjective experience results

**Figure 6:**
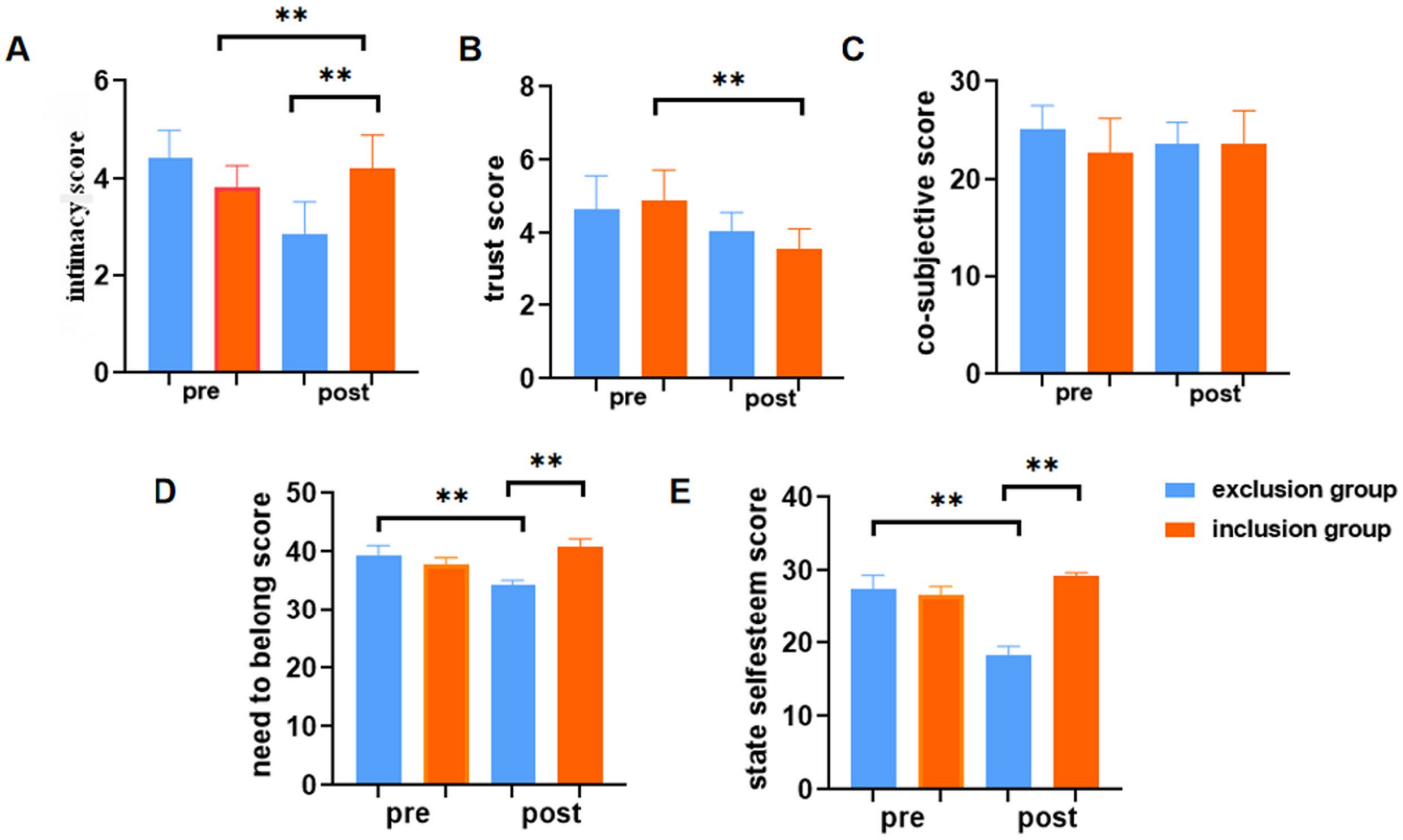
Comparison of subjective-experience indices. **(A)** intimacy score; **(B)** trust score; **(C)** subjective cooperation score; **(D)** need for belonging score; **(E)** state self-esteem score. Error bars represent standard error; ** indicates *p* < 0.01.

To examine changes in participants’ subjective feelings before and after social exclusion and verify the validity of the social exclusion manipulation, post-experiment questionnaires were administered to assess intimacy (Figure 6A), trust (Figure 6B), subjective cooperation (Figure 6C), need to belong (Figure 6D), and state self-esteem (Figure 6E). The descriptive statistics for subjective experience in Supplementary Table 2.

A 2 (group: exclusion group vs. inclusion group) × 2 (time: pre-test vs. post-test) repeated-measures analysis of variance was performed to analyze the differences in these subjective indicators (see Table 1).

**Table 1:** ANOVA results for subjective-experience indices.

Effect	Index	*F*	*p*	*η*_p_^2^
Main effect of time	Intimacy	3.88	0.055	0.08
	Trust	0.27	0.605	0.01
	Subjective cooperativeness	0.88	0.353	0.02
	Need to belong	1.02	0.319	0.02
	State self-esteem	17.55	0.001	0.28
Time × group interaction effect	Intimacy	10.48	0.002	0.19
	Trust	3.13	0.083	0.06
	Subjective cooperativeness	0.82	0.369	0.02
	Need to belong	12.30	0.001	0.21
	State self-esteem	52.71	0.001	0.53
Main effect of group	Intimacy	2.02	0.162	0.04
	Trust	7.69	0.008	0.14
	Subjective cooperativeness	0.09	0.765	0.00
	Need to belong	4.54	0.038	0.09
	State self-esteem	15.32	0.001	0.25

The results revealed significant main effects of time and group on state self-esteem scores. Significant interaction effects of time and group were observed for intimacy, need to belong, and state self-esteem. No significant effects were found for the remaining indicators.

Simple effect analyses with false discovery rate (FDR) correction were further conducted for indicators with significant main and interaction effects, and the results were as follows (Figure 6):

In the exclusion group, significant temporal changes were found in the need to belong [,*t*_(22)_ = 3.82, *p* = 0.008, Cohen’s *d* = 0.55] and state self-esteem [*t*_(22)_ = 8.40, *p* = 0.001, Cohen’s *d* = 1.18], with significantly lower scores after social exclusion relative to the baseline level. In contrast, the inclusion group showed a significant increase in intimacy scores after the inclusion manipulation [*t*_(22)_ = −3.11, *p* = 0.005, Cohen’s *d* = 0.46].

For the pre-test measurement, there were no significant group differences in intimacy, trust, need to belong, and state self-esteem (all *p* > 0.05), indicating equivalent baseline subjective levels between the two groups. For the post-test measurement, however, significant group differences were observed across all subjective indicators. Specifically, the exclusion group exhibited significantly lower scores than the inclusion group in intimacy [*t*_(22)_ = −3.11, *p* = 0.005, Cohen’s *d* = 0.46], trust [*t*_(22)_ = −3.01, *p* = 0.006, Cohen’s *d* = 0.44], need to belong [*t*_(22)_ = −3.82, *p* = 0.008, Cohen’s *d* = 0.55], and state self-esteem [*t*_(22)_ = −8.40, *p* = 0.001, Cohen’s *d* = 1.18].

These findings demonstrate that face-to-face social exclusion exerts extensive negative impacts on individuals’ subjective experiences, including multiple interpersonal indicators of intimacy, trust, need to belong, and state self-esteem. In the post-test stage, participants in the exclusion group reported significantly lower interpersonal and self-perception scores than those in the inclusion group. This indicates that social exclusion impairs individuals’ interpersonal connection and sense of belonging, while reducing interpersonal trust and situational state self-esteem. See Figure 6 for detailed results.

### 3.3 Behavioral results

The descriptive statistics for behavioral measures in Supplementary Table 3. To examine the immediate impact of social exclusion on interpersonal cooperation, we conducted a 2 (Group: exclusion vs. inclusion) × 2 (Time: pre-test vs. post-test) repeated-measures ANOVA on the behavioral indices collected during the Prisoner’s Dilemma task (see Table 2 for details).

**Table 2:** ANOVA for behavioral indices.

Effect	Index	*F*	*p*	*η*_p_^2^
Main effect of time	Average cooperation rate	0.32	0.576	0.01
	Average defection rate	0.05	0.824	0.00
	Average cooperation RT	0.15	0.701	0.01
	Average defection RT	0.84	0.369	0.04
	Cooperation efficiency	0.29	0.598	0.01
	Defection efficiency	0.61	0.444	0.03
Time ×group interaction effect	Average cooperation rate	0.65	0.429	0.03
	Average defection rate	1.35	0.259	0.06
	Average cooperation RT	1.91	0.181	0.08
	Average defection RT	6.88	0.016	0.24
	Cooperation efficiency	0.35	0.562	0.02
	Defection efficiency	4.54	0.044	0.17
Main effect of group	Average cooperation rate	0.14	0.717	0.01
	Average defection rate	0.18	0.672	0.01
	Average cooperation RT	1.11	0.304	0.05
	Average defection RT	0.01	0.909	0.00
	Cooperation efficiency	0.17	0.684	0.01
	Defection efficiency	0.01	0.922	0.00

**Figure 7:**
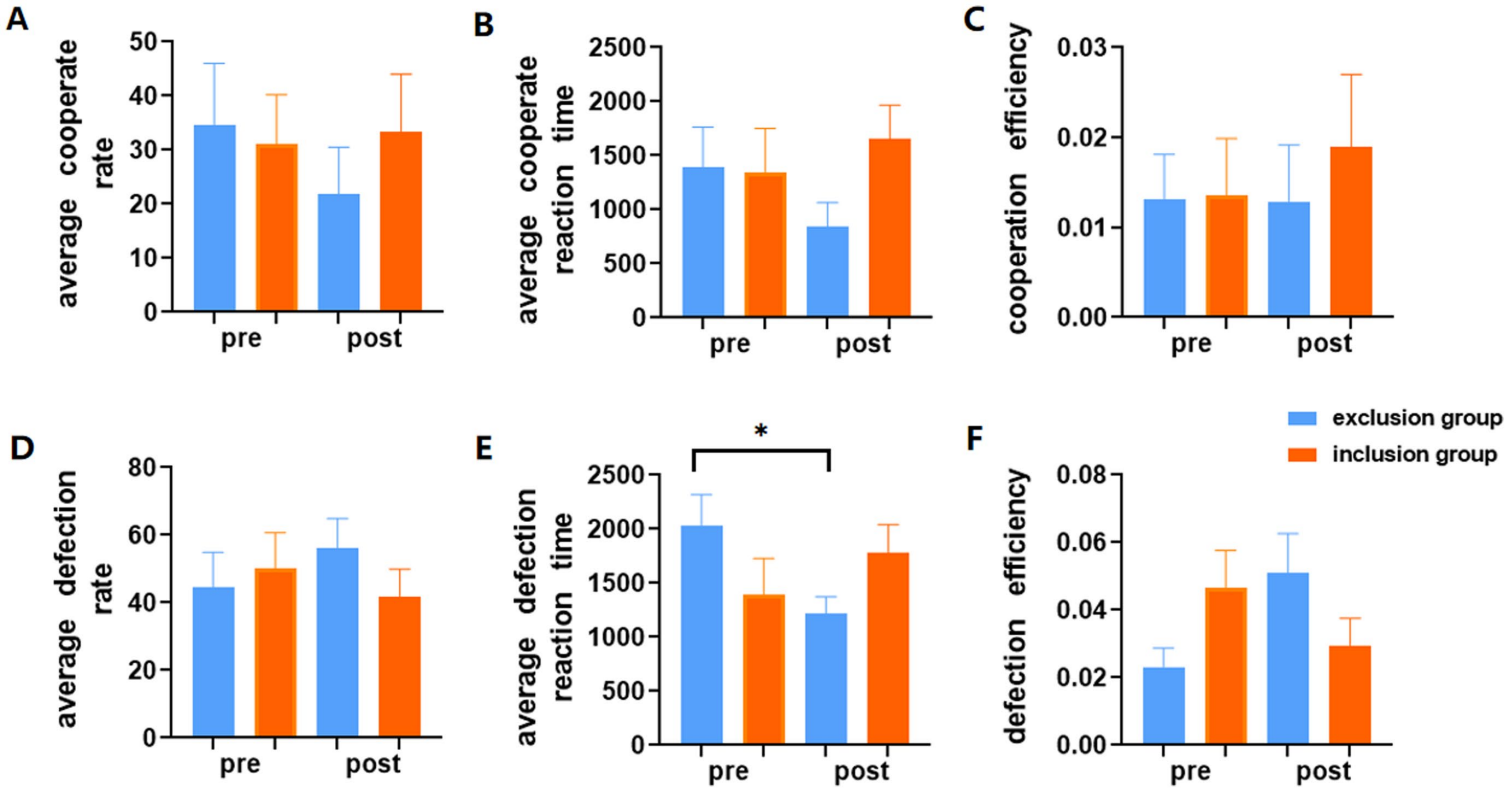
Comparison of behavioral indices. **(A)** average cooperation rate; **(B)** average cooperation reaction time; **(C)** cooperation efficiency; **(D)** average defection rate; **(E)** average defection reaction time; **(F)** defection efficiency. Error bars represent standard error; * indicates *p* < 0.05.

Neither the main effect of Time nor the main effect of Group reached significance for average cooperation rate (Figure 7A), average defection rate (Figure 7D), average cooperation reaction time (RT; Figure 7B), average defection RT (Figure 7E), cooperation efficiency (Figure 7C), or defection efficiency (Figure 7F, *p* > 0.05), indicating that these behavioral measures did not differ across time points or between groups.

However, significant Time × Group interactions were observed for mean defection RT (*p* = 0.016) and defection efficiency (*p* = 0.044); all other interaction terms (cooperation rate, defection rate, cooperation RT, cooperation efficiency) were non-significant (*p* > 0.05). These findings suggest that the temporal change in defection RT and defection efficiency differed between the exclusion and inclusion groups, whereas the remaining indices showed no reliable group- or time-related differences.

For the two indices showing a significant interaction, follow-up simple-effects analyses were conducted in Figure 7.

The results showed that the exclusion group exhibited a significant pretest-posttest difference only in average defection reaction time [*t*_(22)_ = −2.50, *p* = 0.020, Cohen’s *d* = 0.36], with significantly faster reaction time in the post-test relative to the pre-test, while the temporal change in defection efficiency was non-significant. By contrast, the inclusion group showed no significant temporal alterations in either defection RT or defection efficiency (reaction time: *p* = 0.240; efficiency: *p* = 0.350), indicating that the social exclusion context accelerated dyadic defection decision-making speed. In addition, no significant group differences were found in average defection reaction time at pre-test (*p* = 0.164) and post-test (*p* = 0.069), as well as in defection efficiency at pre-test (*p* = 0.145) and post-test (*p* = 0.170). Collectively, these findings suggest that social exclusion significantly shortens the reaction time of defection decisions and facilitates the emergence of individual defection behaviors.

### 3.4 INS results during cooperative decision-making

The descriptive statistics for INS during cooperative decision-making in Supplementary Table 4. To explore alterations in INS during interpersonal decision-making under social exclusion, a 2 (Group: exclusion vs. inclusion) × 2 (Time: pre-test vs. post-test) repeated-measures ANOVA was performed on INS data (see Supplementary Table 5). Significant Time × Group interaction effects were observed in CH3 and CH6. Significant main effects of time were found in CH6, CH11, CH12, CH19, and CH22, while significant main effects of group were identified in CH9, CH14, and CH17.

**Figure 8:**
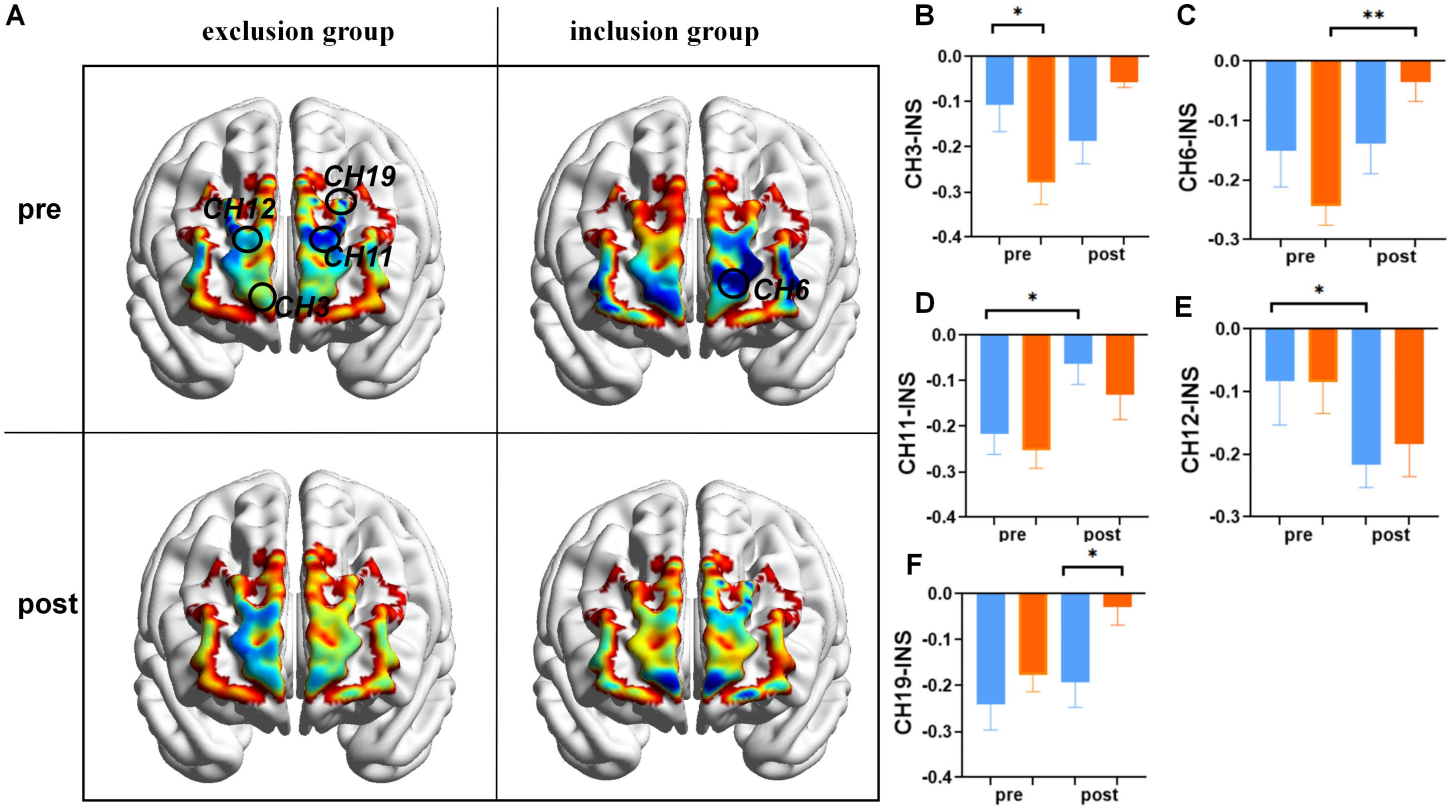
Comparison of INS indices during cooperative decision-making. **(A)** INS maps of the two groups under different experimental conditions. Compared with before exclusion, the exclusion group showed significantly decreased INS levels in CH3 and CH12, and significantly increased INS level in CH11 during cooperative decision-making after social exclusion. Compared with before inclusion, the inclusion group exhibited a significantly elevated INS level in CH6 during cooperative decision-making after inclusion. In CH19, the post-exclusion INS level of the exclusion group was significantly lower than the post-inclusion INS level of the inclusion group. The INS alteration magnitude of CH3 in the exclusion group was significantly smaller than that in the inclusion group. **(B)** INS levels of CH3 in the two groups under different tasks. **(C)** INS levels of CH6 in the two groups under different tasks. **(D)** INS levels of CH11 in the two groups under different tasks. **(E)** INS levels of CH12 in the two groups under different tasks. **(F)** INS levels of CH19 in the two groups under different tasks. Error bars represent standard error; * indicates *p* < 0.05; ** indicates *p* < 0.01.

Further simple effect analyses and post-hoc tests were conducted for all channels with significant main and interaction effects (see Figure 8A). The results were as follows:

In the exclusion group, post-exclusion INS was significantly decreased in CH12 [Figure 8E, right frontopolar area; *t*_(22)_ = 2.21, *p* = 0.037, Cohen’s *d* = 0.32] and significantly increased in CH11 [Figure 8D, left frontopolar area; *t*_(22)_ = −2.61, *p* = 0.016, Cohen’s *d* = 0.36] compared with the pre-exclusion baseline. In the inclusion group, significantly elevated post-inclusion INS values were observed in CH6 [Figure 8C, left frontopolar area; *t*_(22)_ = −4.10, *p* = 0.001, Cohen’s *d* = 0.59] and CH19 [*T*_(22)_ = −3.55, *p* = 0.002, Cohen’s *d* = 0.51], whereas no significant temporal change was found in CH22 [*t*_(22)_ = −1.86, *p* = 0.075, Cohen’s *d* = 0.27]. These findings indicated that social inclusion and social exclusion exert channel-specific modulatory effects on prefrontal INS during cooperative decision-making.

In terms of group differences, CH6 showed no significant inter-group differences at either pre-test (*p* = 0.189) or post-test (*p* = 0.095). For CH19 (left frontal eye field) (Figure 8F), the inter-group difference was non-significant at pre-test (*p* = 0.351) but became significant at post-test (*p* = 0.024), with the exclusion group exhibiting lower INS than the inclusion group after the experimental manipulation.

Additionally, significant baseline group differences were found in CH3 (Figure 8B, *p* = 0.034), CH9 (*p* = 0.013), CH14 (*p* = 0.003), and CH17 (*p* = 0.008). To eliminate the confounding influence of baseline disparities, change scores (post-test minus pre-test) were calculated as corrected indicators, and independent-samples t-tests were performed on these delta values. The results revealed a significant group difference only in the CH3 change score [right orbitofrontal cortex; *t*_(22)_ = −3.62, *p* = 0.002, Cohen’s *d* = 3.79], such that the magnitude of INS alteration during cooperative decision-making was significantly smaller in the exclusion group than in the inclusion group.

### 3.5 Correlation analysis during cooperative decision-making

To explore whether changes in cooperative behaviors following social exclusion were associated with alterations in inter-brain synchronization and subjective feelings during cooperative decision-making, the pre-test values of behavioral indicators, INS data, and subjective questionnaire scores in the exclusion group were adopted as baseline levels. Change scores (post-test minus pre-test) were calculated for all indicators and subjected to Pearson correlation analysis.

The results demonstrated that the change score of average cooperation reaction time was significantly and negatively correlated with the change score of trust level (*r* = −0.75, *p* = 0.005), indicating that increased trust corresponds to faster cooperative decision-making.

### 3.6 Psychological mediating effect analysis of social exclusion on behavior during cooperative decision-making

To reveal the underlying psychological mechanism of how social exclusion affects behavioral performance in cooperative decision-making, this study adopted the PROCESS macro Model 4 to conduct a simple mediating effect analysis (5,000 Bootstrap resamples, 95% confidence intervals).

In the mediation model, social exclusion was set as the independent variable, behavioral indicators during cooperative decision-making (average cooperation rate, average cooperation reaction time, and cooperation efficiency) were defined as dependent variables, and subjective experience indicators (intimacy, trust, subjective cooperation, need to belong, and state self-esteem) were selected as mediating variables, with a valid sample size of N = 24.

The results showed that no significant mediating effects were identified in the cooperative decision-making context.

### 3.7 INS results during the defection decision-making

The descriptive statistics for INS during defection decision-making in Supplementary Table 6. To investigate changes in INS during interpersonal defection decision-making under social exclusion, a 2 (Group: exclusion vs. inclusion) × 2 (Time: pre-test vs. post-test) repeated-measures ANOVA was conducted on the collected INS indicators. The results are presented in Supplementary Table 7. Significant Time × Group interaction effects were found in CH15 and CH20. Significant main effects of time were observed in CH11 and CH16, while a significant main effect of group was identified in CH6.

**Figure 9:**
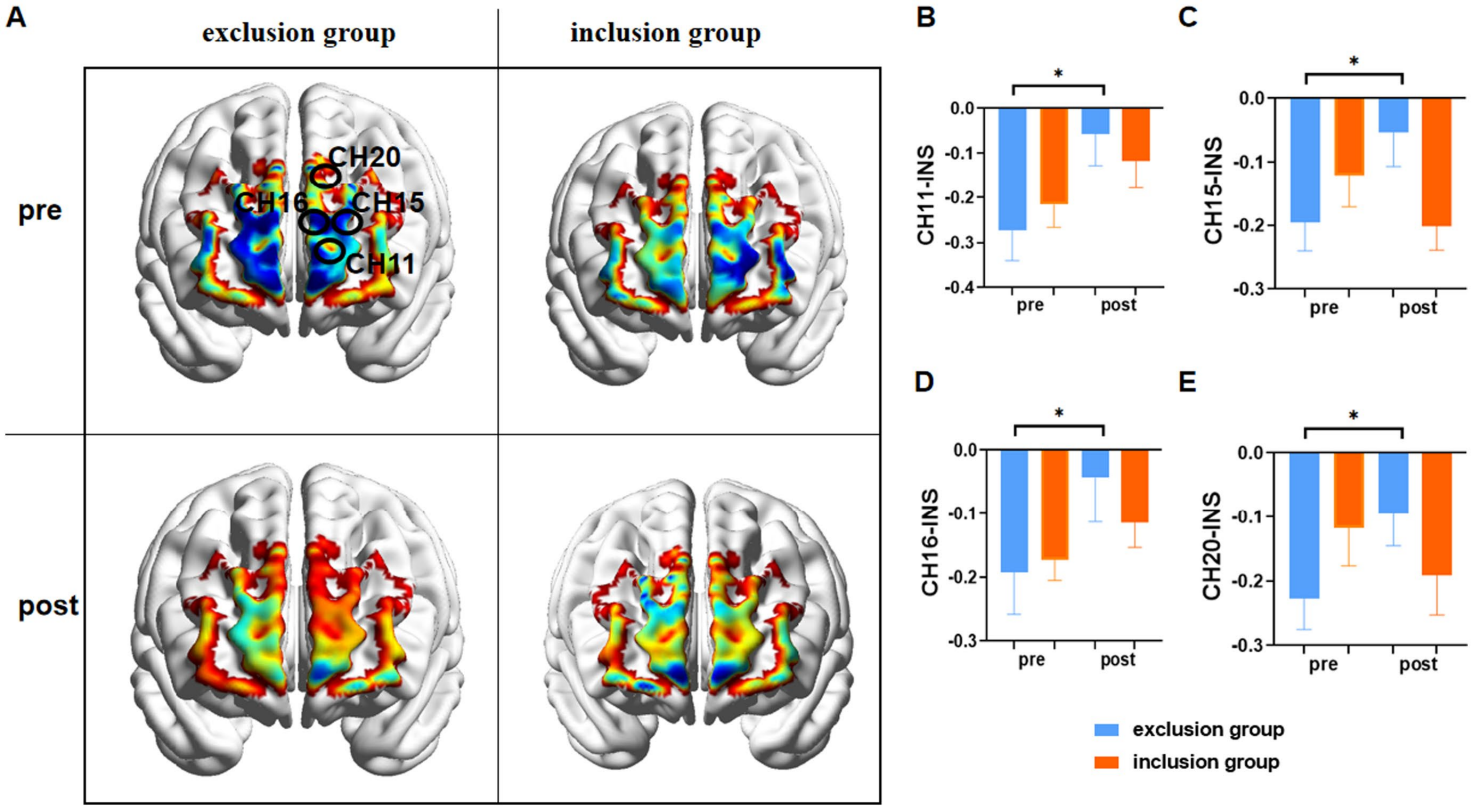
**(A)** INS maps of the two groups under different experimental conditions. Compared with before exclusion, the INS of CH11, CH15, CH16, and CH20 was significantly enhanced in the exclusion group during defection decision-making after social exclusion. **(B)** INS levels of CH11 in the two groups under different tasks. **(C)** INS levels of CH15 in the two groups under different tasks. **(D)** INS levels of CH16 in the two groups under different tasks.(E) INS levels of CH20 in the two groups under different tasks. Note: Error bars represent standard error; * indicates *p* < 0.05.

Further simple effect analyses and post-hoc tests were performed for all channels with significant main and interaction effects. The results showed that the exclusion group exhibited significant temporal increases in INS after social exclusion in multiple channels (see Figure 9A), including CH11 [Figure 9B, left frontopolar area; *t*_(22)_ = −2.33, *p* = 0.030, Cohen’s *d* = 0.34], CH15 [Figure 9C, left dorsolateral prefrontal cortex; *t*_(22)_ = −2.09, *p* = 0.049, Cohen’s *d* = 0.30), CH16 (Figure 9D, left dorsolateral prefrontal cortex; *t*_(22)_ = −2.13, *p* = 0.045, Cohen’s *d* = 0.31], and CH20 [Figure 9E, right frontal eye field; *t*_(22)_ = −2.22, *p* = 0.038, Cohen’s *d* = 0.32]. In contrast, the inclusion group showed no significant temporal changes in INS across all measured channels (*p* > 0.05).

For CH15 (left dorsolateral prefrontal cortex), no significant group difference was observed at pre-test (*p* = 0.285), whereas a significant group difference emerged at post-test [*t*_(22)_ = 2.27, *p* = 0.033, Cohen’s *d* = 0.97], with the exclusion group showing significantly higher INS than the inclusion group after the experimental manipulation. A significant baseline group difference was detected in CH6 (*p* = 0.025). To eliminate the confounding effect of baseline disparities, change scores (post-test minus pre-test) were calculated and analyzed via independent-samples t-tests. The results indicated no significant group difference in the CH6 change score (*p* = 0.550). However, a significant group difference was found for the CH15 change score [*t*_(22)_ = −2.31, *p* = 0.031, Cohen’s *d* = 0.98], revealing that the INS alteration magnitude of CH15 in the exclusion group was significantly greater than that in the inclusion group.

### 3.8 Correlational analysis during defection decision-making

To further examine whether changes in indicators of defection following social exclusion were associated with alterations in INS during defection decision-making, pre-test behavioral and INS data of the exclusion group were taken as the baseline. Change scores (post-test minus pre-test) were calculated as evaluation indicators, and Pearson correlation analysis was performed between the behavioral change scores and INS change scores.

The results revealed a significant positive correlation between the change score of defection efficiency and the INS change score of CH11 (*r* = 0.67, *p* = 0.018), indicating that increased INS in CH11 (left frontopolar area) was accompanied by enhanced defection decision-making behaviors.

### 3.9 Mediating effect analysis of social exclusion on behavior during defection decision-making

To explore the potential psychological mechanism underlying the effect of social exclusion on defection behavior, this study performed a simple mediation analysis using PROCESS Macro Model 4 with 5,000 Bootstrap resamples and 95% confidence intervals (CIs). In the mediation model, social exclusion was defined as the independent variable, behavioral indicators of defection decision-making (average defection rate, average defection reaction time, and defection efficiency) were set as dependent variables, and subjective experience indicators (intimacy, trust, subjective cooperation, need to belong, and state self-esteem) were selected as mediating variables, with a valid sample size of *N* = 24.

The results indicated that a significant mediating effect existed only when average defection reaction time served as the dependent variable and state self-esteem served as the mediating variable. Social exclusion significantly predicted state self-esteem with a well-fitted model (*R*^2^ = 0.66, *F*_(1,22)_ = 42.69, *p* < 0.001). The regression coefficient was significant (*b* = 11.63, *t*_(22)_ = 6.53, *p* < 0.001), indicating that social exclusion significantly reduced individuals’ state self-esteem.

The total effect of social exclusion on defection reaction time was significant (*b* = −1204.05, *t*_(22)_ = −2.62, *p* = 0.015, 95% CI [−2156.06, −252.05]), with a standardized coefficient of −0.96. After controlling for the mediating variable, the direct effect of social exclusion on defection reaction time remained significant (*b* = −2454.33, *t*_(21)_ = −3.35, *p* = 0.003, 95% CI [−3977.27, −931.39]), with a standardized coefficient of −1.95.

The indirect effect of state self-esteem between social exclusion and defection reaction time was significant (indirect effect = 1250.27, Boot SE = 485.45, 95% CI [285.41, 2221.11]), and the confidence interval did not contain zero. The partially standardized indirect effect was 0.99, confirming a valid mediating effect. Specifically, state self-esteem played a partial mediating role in the association between social exclusion and defection reaction time.

## 4 Discussion

Adopting the fNIRS hyperscanning technique with a randomized controlled design, the present study systematically investigated the effects of face-to-face social exclusion on interpersonal cooperation and defection behaviors among college students, and further revealed the underlying psychological and neural mechanisms from the perspectives of subjective experience, behavioral performance, and interpersonal neural synchronization (INS). The results demonstrated that face-to-face social exclusion significantly reduced individuals’ feelings of intimacy, trust, need to belong, and state self-esteem. At the behavioral level, social exclusion significantly shortened the reaction time of defection decision-making and accelerated defection decision speed. At the neural level, channel-specific alterations in prefrontal INS were observed during both cooperation and defection tasks. Correlation analysis revealed that trust level was significantly negatively correlated with cooperation reaction time, and defection efficiency was significantly positively correlated with INS in the left frontopolar area (CH11). Mediation analysis further indicated that subjective experience partially mediated the relationship between social exclusion and defection decision speed. Overall, these findings suggest that face-to-face social exclusion accelerates defection decision-making by impairing core subjective experiences and reshaping prefrontal inter-brain coordination patterns. This study comprehensively elucidates the mechanism of social exclusion with high ecological validity from integrated psychological, behavioral, and neural perspectives.

Existing studies have indicated that social exclusion reduces prosocial behaviors, increases defensive responses, and exerts sustained negative impacts on interpersonal interactions (Twenge et al., 2001). Consistent with the core tenets of the belongingness needs theory, the present study found that social exclusion significantly undermined individuals’ feelings of intimacy, trust, need to belong, and state self-esteem, suggesting that social exclusion directly threatens fundamental socio-psychological needs and further alters individuals’ subsequent interpersonal decision-making tendencies. At the behavioral level, no significant group difference was observed in cooperation rate, whereas social exclusion significantly accelerated defection decision-making speed. This finding indicates that the influence of social exclusion on interpersonal behavior is not reflected in the tendency to cooperate or defect, but rather in decision-making speed and efficiency. This result supports the social monitoring system theory, which proposes that individuals enhance social vigilance and make defensive choices more rapidly after experiencing social exclusion to avoid subsequent social harm (Twenge et al., 2007; Twenge et al., 2002). Furthermore, faster defection decision-making reflects a tendency toward “defensive self-interest,” such that individuals tend to adopt self-protective strategies promptly in negative social contexts (Leary, 2003).

The present study found no significant group difference in overall cooperation rates, which is not entirely consistent with findings from some traditional social exclusion studies. Rather than being contradictory, this result indicates that the influence of social exclusion on cooperative behavior is task-specific and process-specific. In repeated interactive Prisoner’s Dilemma games, participants strategically adjust their decisions based on their partner’s behaviors, leading to comparable overall cooperation rates across groups. Nevertheless, at the internal cognitive decision-making level, social exclusion significantly altered decision processing speed. This suggests that the impact of social exclusion is manifested more in cognitive processing and decision dynamics than in overt behavioral choices. The present findings broaden the understanding of the association between social exclusion and interpersonal cooperation, highlighting the crucial value of temporal decision-making indicators.

In terms of psychological mechanisms, this study revealed that subjective experience partially mediated the relationship between social exclusion and defection decision speed. Social exclusion directly accelerated defection reaction time and indirectly facilitated defection decision-making by impairing individuals’ subjective feelings, demonstrating that subjective psychological experience serves as a critical intermediate pathway linking social exclusion to interpersonal behaviors. This result aligns with the belongingness needs theory, which posits that frustrated belonging needs reduce individuals’ motivation to maintain positive interpersonal interactions and prompt them to adopt defensive defection behaviors more rapidly. In addition, correlation analyses showed that higher trust levels predicted faster cooperative decision-making, and higher defection efficiency was accompanied by enhanced INS in CH11 (left frontopolar area). These findings further indicate that trust, subjective experience, and neural activity collectively constitute an integrated mechanism underlying the influence of social exclusion on interpersonal decision-making.

At the neural mechanism level, the present study identified channel-specific alterations in prefrontal INS during cooperative and defection decision-making. During cooperative interactions, the exclusion group exhibited a significantly lower magnitude of INS changes in the right orbitofrontal cortex (CH3) relative to the acceptance group. The orbitofrontal cortex is critically involved in social value evaluation, emotional processing, and interpersonal decision-making. The reduced INS in this region may reflect diminished cooperative motivation and lowered interpersonal expectation following social exclusion (Eisenberger et al., 2003). During defection decision-making, the exclusion group showed significantly elevated INS in CH15 (left dorsolateral prefrontal cortex), and the INS value of CH11 (left frontopolar area) was significantly positively correlated with defection efficiency. The frontopolar area and dorsolateral prefrontal cortex are core brain regions responsible for social inference, intentional processing, and strategic control (Masten et al., 2009). The enhanced INS in these channels indicates that dyads in the social exclusion context entered a highly vigilant and monitoring-oriented antagonistic interactive state, rather than maintaining cooperative inter-brain collaboration.

One of the most important findings of the present study is that increased INS during defection contexts does not represent interpersonal cooperation, but reflects inter-brain synchronization under high-conflict and high-monitoring interactive states. Traditional hyperscanning studies generally interpret enhanced INS as a marker of positive interpersonal coordination, whereas the current study demonstrates the dual functionality of INS: it can index not only cooperative collaboration but also mutual monitoring during conflict-based game interactions (Liu et al., 2023). After experiencing social exclusion, individuals become more sensitive to others’ intentions and exhibit heightened distrust. Accordingly, they invest more cognitive resources in inferring partners’ intentions and calculating strategic responses during decision-making, which ultimately manifests as elevated prefrontal INS. This viewpoint is consistent with the social brain hypothesis, which proposes that one core function of the prefrontal cortex is to support complex and high-risk social interactions. The present study extends the functional interpretation of INS from a pure “cooperation marker” to a broader “indicator of interactive intensity and depth,” providing a vital theoretical supplement for future hyperscanning research.

Although this study systematically revealed the psychological-behavioral-neural mechanisms underlying face-to-face social exclusion, several limitations remain. First, the relatively limited sample size restricted the in-depth exploration of the moderating effects of individual differences such as sex and personality. Future research could expand the sample size to further examine these moderating roles. Meanwhile, we acknowledge the inherent limitations of our strict sampling design. Although the adoption of same-sex dyads and only-child participants improves the internal validity of the experiment by controlling confounding variables, it inevitably reduces the generalizability of the research findings. Future studies may relax the sampling criteria by recruiting cross-sex dyads and participants with diverse family structures and sports backgrounds, so as to further verify the stability and universality of the current conclusions. Second, this study only adopted aggregated pre- and post-test behavioral indicators and failed to deeply explore trial-by-trial strategy fluctuations, reciprocal patterns, and dynamic feedback adjustments. Combining trial-by-trial dynamic analysis and computational modeling in future research can help further clarify how distinct decision-making strategies and feedback adaptation mediate the effects of social exclusion on cooperative and defective behaviors. Third, this study only employed a single face-to-face rejection paradigm. Subsequent studies could set neutral control groups and experimental groups with different levels of exclusion intensity to better isolate context-specific effects.

In conclusion, the present study confirms that face-to-face social exclusion accelerates defection decision-making and impairs interpersonal cooperative motivation by disrupting core subjective experiences, altering prefrontal inter-brain neural synchronization, and exerting a partial mediating effect through subjective feelings. From a high ecological validity perspective, these findings enrich the interpersonal neural mechanism of social exclusion and provide solid theoretical and empirical evidence for understanding campus social conflicts and improving interpersonal adaptation among college students.

## 5 Conclusion

Adopting the fNIRS hyperscanning technique and a face-to-face social exclusion paradigm combined with the Prisoner’s Dilemma task, this study systematically investigated the effects of social exclusion on interpersonal cooperation and defection behaviors among college students from three dimensions: subjective experience, behavioral performance, and inter-brain neural synchronization (INS). The results revealed that face-to-face social exclusion significantly reduced individuals’ intimacy, trust, need to belong, and state self-esteem. At the behavioral level, social exclusion primarily accelerated the speed of defection decision-making rather than altering the overall cooperation rate. At the neural level, channel-specific INS alterations were observed in the prefrontal cortex. Furthermore, correlation and mediation analyses demonstrated that cooperation reaction time was significantly correlated with trust level, and defection efficiency was significantly associated with INS in the left frontopolar area (CH11). Subjective experience also exerted a partial mediating effect on the relationship between social exclusion and defection decision speed. Collectively, these findings indicate that social exclusion accelerates defensive defection decision-making by impairing subjective psychological experiences and reshaping prefrontal inter-brain coordination patterns, providing high ecological validity evidence for the psychological and neural mechanisms underlying interpersonal social exclusion.

## Data Availability

The original contributions presented in the study are included in the article/Supplementary material, further inquiries can be directed to the corresponding author.
