# Supplementary material for: The impact of face-to-face social exclusion on university students’ interpersonal cooperation behavior: a hyperscanning study based on fNIRS
Source: Front Neurosci. 2026 May 25;20:1807205. doi: 10.3389/fnins.2026.1807205 (PMC13243252; doi:10.3389/fnins.2026.1807205)
Supplement: Supplementary file 1 [file Data_Sheet_1.docx]

**Supplement**

**1. Anatomical brain-region labels**

S-Table1 Brain Region Locations

|  |  | mni coordinate | | | Brodman Talairach | |
| --- | --- | --- | --- | --- | --- | --- |
|  |  | X | Y | Z | Area | Percentage |
| CH1 | 23 | -40 | 58 | -13 | 10 Frontopolar | 0.27642 |
|  |  |  |  |  | 11 Orbitofrontal | 0.71545 |
|  |  |  |  |  | 47 Inferior prefrontal gyrus, | 0.0081301 |
| CH 2 | 24 | -13 | 70 | -12 | 10 Frontopolar | 0.22569 |
|  |  |  |  |  |  |  |
|  |  |  |  |  | 11 Orbitofrontal | 0.77431 |
|  |  |  |  |  |  |  |
| CH 3 | 25 | 16 | 70 | -13 | 10 Frontopolar | 0.21379 |
|  |  |  |  |  | 11 Orbitofrontal | 0.78621 |
|  |  |  |  |  |  |  |
| CH 4 | 26 | 40 | 62 | -13 | 10 Frontopolar | 0.34274 |
|  |  |  |  |  | 11 Orbitofrontal | 0.65726 |
| CH 5 | 27 | -51 | 47 | -3 | 10 Frontopolar | 0.24731 |
|  |  |  |  |  | 11 Orbitofrontal | 0.0071685 |
|  |  |  |  |  | 46 Dorsolateral prefrontal cortex | 0.10753 |
|  |  |  |  |  | 47 Inferior gyrus, | 0.63799 |
| CH 6 | 28 | -30 | 67 | 1 | 10 Frontopolar | 1 |
|  |  |  |  |  |  |  |
|  |  |  |  |  |  |  |
|  |  |  |  |  |  |  |
| CH 7 | 29 | 1 | 69 | 3 | 10 Frontopolar | 0.9967 |
|  |  |  |  |  |  |  |
|  |  |  |  |  | 11 Orbitofrontal | 0.0033003 |
|  |  |  |  |  |  |  |
| CH 8 | 30 | 31 | 68 | 0 | 10 Frontopolar | 1 |
|  |  |  |  |  |  |  |
|  |  |  |  |  |  |  |
|  |  |  |  |  |  |  |
| CH 9 | 31 | 49 | 53 | -5 | 10 Frontopolar | 0.54924 |
|  |  |  |  |  | 11 Orbitofrontal | 0.10985 |
|  |  |  |  |  | 46 Dorsolateral prefrontal cortex | 0.0075758 |
|  |  |  |  |  | 47 Inferior prefrontal gyrus, | 0.33333 |
| CH 10 | 32 | -42 | 55 | 18 | 10 Frontopolar | 0.80162 |
|  |  |  |  |  | 46 Dorsolateral prefrontal | 0.19838 |
| CH 11 | 33 | -14 | 70 | 20 | 10 Frontopolar | 1 |
|  |  |  |  |  |  |  |
| CH 12 | 34 | 17 | 71 | 19 | 10 Frontopolar | 1 |
|  |  |  |  |  |  |  |
|  |  |  |  |  |  |  |
| CH 13 | 35 | 43 | 60 | 12 | 10 Frontopolar | 0.93651 |
|  |  |  |  |  | 46 Dorsolateral prefrontal cortex, | 0.063492 |
| CH 14 | 36 | -50 | 34 | 30 | 9 Dorsolateral prefrontal cortex, | 0.18045 |
|  |  |  |  |  | 45 pars triangularis Broca's | 0.0037594 |
|  |  |  |  |  | 46 Dorsolateral prefrontal cortex, | 0.81579 |
| CH 15 | 37 | -29 | 53 | 35 | 9 Dorsolateral prefrontal cortex, | 0.72197 |
|  |  |  |  |  | 10 Frontopolar | 0.27803 |
| CH 16 | 38 | -2 | 60 | 36 | 9 Dorsolateral prefrontal cortex, | 0.62082 |
|  |  |  |  |  | 10 Frontopolar | 0.37918 |
|  |  |  |  |  |  |  |
| CH 17 | 39 | 30 | 57 | 32 | 9 Dorsolateral prefrontal cortex, | 0.5 |
|  |  |  |  |  | 10 Frontopolar | 0.5 |
| CH 18 | 40 | 51 | 42 | 24 | 10 Frontopolar | 0.11069 |
|  |  |  |  |  | 46 Dorsolateral prefrontal cortex, | 0.88931 |
| CH 19 | 41 | -38 | 33 | 47 | 8 Includes Frontal eyefields, | 0.74222 |
|  |  |  |  |  | 9 Dorsolateral prefrontal cortex, | 0.25778 |
| CH 20 | 42 | -13 | 49 | 49 | 8 Includes Frontal eyefields, | 0.78481 |
|  |  |  |  |  | 9 Dorsolateral prefrontal cortex, | 0.21519 |
| CH 21 | 43 | 17 | 52 | 45 | 8 Includes Frontal eyefields, | 0.48333 |
|  |  |  |  |  | 9 Dorsolateral prefrontal cortex, | 0.51667 |
| CH 22 | 44 | 42 | 37 | 42 | 8 Includes Frontal eyefields, | 0.36111 |
|  |  |  |  |  | 9 Dorsolateral prefrontal cortex, | 0.62037 |
|  |  |  |  |  | 46 Dorsolateral prefrontal cortex, | 0.018519 |

**2 Descriptive Statistics for Subjective Experience**

S-Table2 Descriptive Statistics for Subjective Experience (M ± SD)

|  |  | Pre | | Post | |
| --- | --- | --- | --- | --- | --- |
| Measures | Group | *M* | *SD* | *M* | *SD* |
| Intimacy(points) | 1 | 4.42 | 0.90 | 2.88 | 1.03 |
|  | 2 | 3.83 | 0.69 | 4.21 | 1.08 |
| Trust(points) | 1 | 4.67 | 1.44 | 4.92 | 1.28 |
|  | 2 | 4.04 | 0.84 | 3.58 | 0.85 |
| Subjective cooperativeness(points) | 1 | 23.67 | 3.36 | 23.63 | 5.33 |
|  | 2 | 25.17 | 3.73 | 22.79 | 5.42 |
| Need to belong(points) | 1 | 39.38 | 5.91 | 34.33 | 2.88 |
|  | 2 | 37.96 | 3.63 | 40.75 | 5.06 |
| State self-esteem(points) | 1 | 27.46 | 6.50 | 18.29 | 4.21 |
|  | 2 | 26.79 | 3.66 | 29.25 | 1.64 |

Note: 1 = exclusion group; 2 = inclusion group; M = mean; SD = standard deviation.

**3 Descriptive Statistics for Behavioral Measures**

S-Table3 Descriptive Statistics for Behavioral Measures (M ± SD)

|  |  | Pre | | Post | |
| --- | --- | --- | --- | --- | --- |
| Measures | Group | *M* | *SD* | *M* | *SD* |
| Mean cooperation rate（%） | 1 | 34.72 | 39.12 | 21.94 | 29.76 |
|  | 2 | 31.11 | 31.44 | 33.33 | 36.95 |
| Mean defection rate（%） | 1 | 44.44 | 36.33 | 56.39 | 29.56 |
|  | 2 | 50.00 | 37.20 | 41.94 | 27.91 |
| Mean cooperation response time（ms） | 1 | 1388.89 | 1300.02 | 838.90 | 787.10 |
|  | 2 | 1343.32 | 1413.93 | 1652.00 | 1093.47 |
| Mean defection response time（ms） | 1 | 2031.49 | 998.96 | 1219.00 | 526.05 |
|  | 2 | 1394.92 | 1159.23 | 1786.48 | 885.49 |
| Cooperation efficiency | 1 | 0.01 | 0.02 | 0.01 | 0.02 |
|  | 2 | 0.01 | 0.02 | 0.02 | 0.03 |
| Defection efficiency | 1 | 0.02 | 0.02 | 0.05 | 0.04 |
|  | 2 | 0.04 | 0.04 | 0.03 | 0.03 |

Note: 1 = exclusion group; 2 = inclusion group; M = mean; SD = standard deviation.

**4 Descriptive Statistics for INS Measures**

S-Table4 Descriptive Statistics for INS Indices During Cooperation（M±SD）

|  |  | Pre | | Post | |
| --- | --- | --- | --- | --- | --- |
| Measures | Group | M | SD | M | SD |
| CH1 | 1 | -0.08 | 0.17 | -0.25 | 0.30 |
|  | 2 | -0.10 | 0.27 | -0.14 | 0.11 |
| CH2 | 1 | -0.15 | 0.12 | -0.18 | 0.14 |
|  | 2 | -0.16 | 0.19 | -0.21 | 0.12 |
| CH3 | 1 | -0.11 | 0.20 | -0.19 | 0.17 |
|  | 2 | -0.28 | 0.17 | -0.06 | 0.04 |
| CH4 | 1 | -0.03 | 0.14 | -0.05 | 0.19 |
|  | 2 | -0.11 | 0.13 | -0.05 | 0.19 |
| CH5 | 1 | -0.07 | 0.20 | -0.10 | 0.19 |
|  | 2 | -0.21 | 0.14 | -0.02 | 0.14 |
| CH6 | 1 | -0.15 | 0.21 | -0.14 | 0.17 |
|  | 2 | -0.24 | 0.11 | -0.04 | 0.11 |
| CH7 | 1 | -0.14 | 0.20 | -0.12 | 0.18 |
|  | 2 | -0.16 | 0.17 | -0.07 | 0.13 |
| CH8 | 1 | -0.16 | 0.15 | -0.21 | 0.16 |
|  | 2 | -0.19 | 0.14 | -0.08 | 0.21 |
| CH9 | 1 | -0.11 | 0.14 | -0.05 | 0.17 |
|  | 2 | -0.28 | 0.17 | -0.21 | 0.19 |
| CH10 | 1 | -0.21 | 0.17 | -0.15 | 0.13 |
|  | 2 | -0.14 | 0.17 | -0.05 | 0.17 |
| CH11 | 1 | -0.22 | 0.15 | -0.06 | 0.16 |
|  | 2 | -0.25 | 0.14 | -0.13 | 0.19 |
| CH12 | 1 | -0.08 | 0.25 | -0.22 | 0.12 |
|  | 2 | -0.08 | 0.18 | -0.19 | 0.18 |
| CH13 | 1 | -0.07 | 0.18 | -0.16 | 0.20 |
|  | 2 | -0.07 | 0.09 | -0.14 | 0.17 |
| CH14 | 1 | -0.35 | 0.18 | -0.25 | 0.13 |
|  | 2 | -0.10 | 0.19 | -0.13 | 0.18 |
| CH15 | 1 | -0.19 | 0.10 | -0.11 | 0.13 |
|  | 2 | -0.11 | 0.22 | -0.14 | 0.08 |
| CH16 | 1 | -0.21 | 0.17 | -0.12 | 0.15 |
|  | 2 | -0.10 | 0.18 | -0.12 | 0.09 |
| CH17 | 1 | -0.23 | 0.14 | -0.19 | 0.19 |
|  | 2 | -0.05 | 0.17 | -0.11 | 0.16 |
| CH18 | 1 | -0.07 | 0.18 | -0.10 | 0.16 |
|  | 2 | -0.15 | 0.16 | -0.14 | 0.26 |
| CH19 | 1 | -0.24 | 0.19 | -0.19 | 0.19 |
|  | 2 | -0.18 | 0.12 | -0.03 | 0.14 |
| CH20 | 1 | -0.21 | 0.18 | -0.16 | 0.14 |
|  | 2 | -0.13 | 0.11 | -0.17 | 0.15 |
| CH21 | 1 | -0.15 | 0.18 | -0.13 | 0.22 |
|  | 2 | -0.06 | 0.16 | -0.12 | 0.17 |
| CH22 | 1 | -0.26 | 0.12 | -0.17 | 0.22 |
|  | 2 | -0.23 | 0.12 | -0.11 | 0.19 |

Note: 1 = exclusion group; 2 = inclusion group; M = mean; SD = standard deviation.

**5 Results of INS During the Cooperative decision**

S-Table5 Results for INS Indices During the Cooperative decision

|  |  | *F* | *p* | *η*_p_^2^ |
| --- | --- | --- | --- | --- |
| main effect of Time | **CH1** | 3.30 | 0.083 | 0.13 |
|  | CH2 | 1.30 | 0.267 | 0.06 |
|  | CH3 | 2.90 | 0.103 | 0.12 |
|  | CH4 | 0.42 | 0.525 | 0.02 |
|  | CH5 | 2.33 | 0.141 | 0.10 |
|  | **CH6** | 9.39 | 0.006 | 0.30 |
|  | CH7 | 1.00 | 0.329 | 0.04 |
|  | CH8 | 0.50 | 0.488 | 0.02 |
|  | CH9 | 2.20 | 0.152 | 0.09 |
|  | **CH10** | 3.34 | 0.081 | 0.13 |
|  | **CH11** | 10.85 | 0.003 | 0.33 |
|  | **CH12** | 7.48 | 0.012 | 0.25 |
|  | CH13 | 2.41 | 0.135 | 0.10 |
|  | CH14 | 0.42 | 0.522 | 0.02 |
|  | CH15 | 0.40 | 0.536 | 0.02 |
|  | CH16 | 0.81 | 0.378 | 0.04 |
|  | CH17 | 0.05 | 0.825 | 0.00 |
|  | CH18 | 0.09 | 0.772 | 0.00 |
|  | **CH19** | 11.03 | 0.003 | 0.33 |
|  | CH20 | 0.03 | 0.867 | 0.00 |
|  | CH21 | 0.23 | 0.636 | 0.01 |
|  | **CH22** | 5.37 | 0.030 | 0.20 |
| Time × Group interaction effect | CH1 | 1.24 | 0.277 | 0.05 |
|  | CH2 | 0.12 | 0.735 | 0.01 |
|  | **CH3** | 13.13 | 0.002 | 0.37 |
|  | CH4 | 1.38 | 0.253 | 0.06 |
|  | **CH5** | 3.97 | 0.059 | 0.15 |
|  | **CH6** | 7.51 | 0.012 | 0.26 |
|  | CH7 | 0.41 | 0.527 | 0.02 |
|  | CH8 | 2.84 | 0.106 | 0.11 |
|  | CH9 | 0.04 | 0.838 | 0.00 |
|  | CH10 | 0.19 | 0.669 | 0.01 |
|  | CH11 | 0.16 | 0.694 | 0.01 |
|  | CH12 | 0.17 | 0.688 | 0.01 |
|  | CH13 | 0.01 | 0.916 | 0.00 |
|  | CH14 | 1.65 | 0.212 | 0.07 |
|  | CH15 | 1.76 | 0.198 | 0.07 |
|  | CH16 | 2.93 | 0.101 | 0.12 |
|  | CH17 | 1.69 | 0.207 | 0.07 |
|  | CH18 | 0.10 | 0.758 | 0.00 |
|  | CH19 | 2.88 | 0.104 | 0.12 |
|  | CH20 | 1.10 | 0.306 | 0.05 |
|  | CH21 | 0.58 | 0.454 | 0.03 |
|  | CH22 | 0.11 | 0.746 | 0.01 |
| main effect of Group | CH1 | 0.35 | 0.563 | 0.02 |
|  | CH2 | 0.15 | 0.707 | 0.01 |
|  | CH3 | 0.18 | 0.678 | 0.01 |
|  | CH4 | 0.52 | 0.478 | 0.02 |
|  | CH5 | 0.51 | 0.484 | 0.02 |
|  | CH6 | 0.01 | 0.920 | 0.00 |
|  | CH7 | 0.12 | 0.736 | 0.01 |
|  | CH8 | 0.91 | 0.351 | 0.04 |
|  | **CH9** | 9.34 | 0.006 | 0.30 |
|  | CH10 | 2.46 | 0.131 | 0.10 |
|  | CH11 | 1.08 | 0.311 | 0.05 |
|  | CH12 | 0.06 | 0.803 | 0.00 |
|  | CH13 | 0.03 | 0.866 | 0.00 |
|  | **CH14** | 16.23 | 0.001 | 0.42 |
|  | CH15 | 0.26 | 0.619 | 0.01 |
|  | CH16 | 1.13 | 0.299 | 0.05 |
|  | **CH17** | 5.60 | 0.027 | 0.20 |
|  | CH18 | 0.91 | 0.349 | 0.04 |
|  | **CH19** | 3.58 | 0.072 | 0.14 |
|  | CH20 | 0.67 | 0.421 | 0.03 |
|  | CH21 | 0.77 | 0.389 | 0.03 |
|  | CH22 | 0.62 | 0.439 | 0.03 |

S-Table6 Descriptive Statistics for INS Indices During defection Decision-Making (M ± SD)

|  |  | Pre | | Post | |
| --- | --- | --- | --- | --- | --- |
| Measures | Group | M | SD | M | SD |
| CH1 | 1 | -0.09 | 0.26 | -0.16 | 0.18 |
|  | 2 | -0.09 | 0.23 | -0.11 | 0.21 |
| CH2 | 1 | -0.12 | 0.08 | -0.23 | 0.18 |
|  | 2 | -0.13 | 0.20 | -0.16 | 0.20 |
| CH3 | 1 | -0.16 | 0.19 | -0.10 | 0.24 |
|  | 2 | -0.21 | 0.17 | -0.05 | 0.15 |
| CH4 | 1 | -0.11 | 0.16 | -0.05 | 0.16 |
|  | 2 | -0.16 | 0.18 | -0.12 | 0.22 |
| CH5 | 1 | -0.14 | 0.25 | -0.09 | 0.18 |
|  | 2 | -0.23 | 0.15 | -0.04 | 0.18 |
| CH6 | 1 | -0.06 | 0.15 | -0.06 | 0.16 |
|  | 2 | -0.18 | 0.10 | -0.13 | 0.18 |
| CH7 | 1 | -0.22 | 0.18 | -0.10 | 0.25 |
|  | 2 | -0.12 | 0.11 | -0.09 | 0.17 |
| CH8 | 1 | -0.16 | 0.20 | -0.15 | 0.21 |
|  | 2 | -0.10 | 0.21 | -0.19 | 0.22 |
| CH9 | 1 | -0.16 | 0.21 | -0.03 | 0.20 |
|  | 2 | -0.23 | 0.17 | -0.13 | 0.27 |
| CH10 | 1 | -0.12 | 0.18 | -0.10 | 0.17 |
|  | 2 | -0.14 | 0.18 | -0.07 | 0.17 |
| CH11 | 1 | -0.27 | 0.23 | -0.06 | 0.25 |
|  | 2 | -0.22 | 0.18 | -0.12 | 0.21 |
| CH12 | 1 | -0.28 | 0.25 | -0.19 | 0.21 |
|  | 2 | -0.12 | 0.11 | -0.26 | 0.15 |
| CH13 | 1 | -0.13 | 0.19 | -0.07 | 0.24 |
|  | 2 | -0.18 | 0.19 | -0.09 | 0.15 |
| CH14 | 1 | -0.21 | 0.22 | -0.18 | 0.15 |
|  | 2 | -0.16 | 0.20 | -0.14 | 0.19 |
| CH15 | 1 | -0.20 | 0.16 | -0.05 | 0.18 |
|  | 2 | -0.12 | 0.17 | -0.20 | 0.13 |
| CH16 | 1 | -0.19 | 0.23 | -0.04 | 0.24 |
|  | 2 | -0.17 | 0.11 | -0.12 | 0.13 |
| CH17 | 1 | -0.19 | 0.14 | -0.16 | 0.21 |
|  | 2 | -0.18 | 0.25 | -0.17 | 0.17 |
| CH18 | 1 | -0.23 | 0.16 | -0.13 | 0.21 |
|  | 2 | -0.17 | 0.23 | -0.10 | 0.21 |
| CH19 | 1 | -0.27 | 0.16 | -0.16 | 0.24 |
|  | 2 | -0.08 | 0.21 | -0.09 | 0.22 |
| CH20 | 1 | -0.23 | 0.16 | -0.10 | 0.17 |
|  | 2 | -0.12 | 0.20 | -0.19 | 0.21 |
| CH21 | 1 | -0.21 | 0.19 | -0.16 | 0.21 |
|  | 2 | -0.17 | 0.23 | -0.19 | 0.21 |
| CH22 | 1 | -0.22 | 0.22 | -0.15 | 0.32 |
|  | 2 | -0.16 | 0.19 | -0.16 | 0.21 |

Note: 1 = exclusion group; 2 = inclusion group; M = mean;SD = standard deviation.

**7.Results of INS During the Defection Decision-Making**

S-Table7 Results for INS Indices During the defection Decision-Making

|  |  | *F* | *p* | *η*_p_^2^ |
| --- | --- | --- | --- | --- |
| main effect of Time | CH1 | 0.60 | 0.449 | 0.03 |
|  | CH2 | 2.18 | 0.154 | 0.09 |
|  | CH3 | 2.68 | 0.116 | 0.11 |
|  | CH4 | 0.92 | 0.348 | 0.04 |
|  | CH5 | 3.68 | 0.068 | 0.14 |
|  | CH6 | 0.19 | 0.665 | 0.01 |
|  | CH7 | 2.00 | 0.171 | 0.08 |
|  | CH8 | 0.56 | 0.462 | 0.03 |
|  | CH9 | 3.61 | 0.071 | 0.14 |
|  | CH10 | 0.71 | 0.409 | 0.03 |
|  | **CH11** | **5.70** | **0.026** | **0.21** |
|  | CH12 | 0.29 | 0.595 | 0.01 |
|  | CH13 | 1.56 | 0.225 | 0.07 |
|  | CH14 | 0.13 | 0.728 | 0.01 |
|  | CH15 | 0.40 | 0.534 | 0.02 |
|  | **CH16** | **4.39** | **0.048** | **0.17** |
|  | CH17 | 0.12 | 0.730 | 0.01 |
|  | CH18 | 0.27 | 0.611 | 0.01 |
|  | CH19 | 0.84 | 0.370 | 0.04 |
|  | CH20 | 0.49 | 0.493 | 0.02 |
|  | CH21 | 0.05 | 0.819 | 0.00 |
|  | CH22 | 0.19 | 0.666 | 0.01 |
| Time × Group interaction effect | CH1 | 0.14 | 0.708 | 0.01 |
|  | CH2 | 0.79 | 0.384 | 0.04 |
|  | CH3 | 0.59 | 0.449 | 0.03 |
|  | CH4 | 0.06 | 0.810 | 0.00 |
|  | CH5 | 1.31 | 0.265 | 0.06 |
|  | CH6 | 0.37 | 0.550 | 0.02 |
|  | CH7 | 0.89 | 0.357 | 0.04 |
|  | CH8 | 0.65 | 0.430 | 0.03 |
|  | CH9 | 0.05 | 0.828 | 0.00 |
|  | CH10 | 0.25 | 0.621 | 0.01 |
|  | CH11 | 0.80 | 0.380 | 0.04 |
|  | CH12 | 4.18 | 0.053 | 0.16 |
|  | CH13 | 0.04 | 0.838 | 0.00 |
|  | CH14 | 0.03 | 0.875 | 0.00 |
|  | **CH15** | **5.35** | **0.031** | **0.20** |
|  | CH16 | 0.84 | 0.369 | 0.04 |
|  | CH17 | 0.04 | 0.851 | 0.00 |
|  | CH18 | 0.07 | 0.801 | 0.00 |
|  | CH19 | 1.26 | 0.274 | 0.05 |
|  | **CH20** | **5.88** | **0.024** | **0.21** |
|  | CH21 | 0.38 | 0.544 | 0.02 |
|  | CH22 | 0.29 | 0.593 | 0.01 |
| main effect of Group | CH1 | 0.19 | 0.671 | 0.01 |
|  | CH2 | 0.30 | 0.589 | 0.01 |
|  | CH3 | 0.00 | 0.976 | 0.00 |
|  | CH4 | 1.57 | 0.223 | 0.07 |
|  | CH5 | 0.22 | 0.642 | 0.01 |
|  | **CH6** | **6.63** | **0.017** | **0.23** |
|  | CH7 | 1.10 | 0.306 | 0.05 |
|  | CH8 | 0.01 | 0.936 | 0.00 |
|  | CH9 | 1.88 | 0.184 | 0.08 |
|  | CH10 | 0.02 | 0.879 | 0.00 |
|  | CH11 | 0.00 | 0.978 | 0.00 |
|  | CH12 | 0.68 | 0.418 | 0.03 |
|  | CH13 | 0.59 | 0.453 | 0.03 |
|  | CH14 | 1.00 | 0.328 | 0.04 |
|  | CH15 | 0.65 | 0.428 | 0.03 |
|  | CH16 | 0.20 | 0.657 | 0.01 |
|  | CH17 | 0.00 | 0.966 | 0.00 |
|  | CH18 | 0.05 | 0.827 | 0.00 |
|  | CH19 | 3.75 | 0.066 | 0.15 |
|  | CH20 | 0.01 | 0.914 | 0.00 |
|  | CH21 | 0.01 | 0.932 | 0.00 |
|  | CH22 | 0.10 | 0.754 | 0.01 |

**8.Questionnaires and Scales**

① Demographic Information Questionnaire

This questionnaire collects participants’ demographic information, including gender, age, psychiatric history, handedness, years of education, normal vision or corrected vision, physical diseases, only-child status, regular exercise habits and main exercise types. By collecting basic information, individuals who fail to meet the experimental inclusion criteria were excluded.

② International Physical Activity Questionnaire-Short Form (IPAQ-SF)

First initiated by Michael Booth in 1996 (Booth, 1996), developed by an international working group on physical activity in 1997, and further revised in 2001 with the support of the World Health Organization and the U.S. Centers for Disease Control and Prevention. The 7-item short-form version was adopted in this study to assess sedentary time, walking, moderate-intensity and vigorous-intensity physical activity. Due to its simplicity and accessibility, IPAQ-SF is widely used to monitor physical activity and ensure physical fitness balance across groups.

Participants reported the weekly frequency and cumulative duration of walking, moderate and vigorous physical activities. Total weekly physical activity was calculated as MET-minutes/week, with the formula:Physical activity level = MET value × weekly frequency (days/week) × daily duration (minutes/day).MET reference values were based on the Chinese Compilation of Physical Activities (CCPA) published by Qiu et al. (2022).

Accumulated evidence has demonstrated that physical activity is closely linked to interpersonal cooperation and socioemotional states. Moderate exercise effectively enhances prosocial tendencies and cooperative willingness, and positively regulates interpersonal interaction. Regular physical activity alleviates negative emotions and buffers adverse psychological responses induced by social exclusion. Accordingly, individual differences in daily physical activity may serve as a confounding variable that interferes with subjective emotions and interpersonal decision-making. To eliminate such confounding effects and ensure baseline homogeneity between paired participants and experimental groups, IPAQ-SF was used to match and balance habitual physical activity levels, improving the validity and robustness of experimental results.

③ Cooperative and Competitive Personality Scale (CCPS)

Developed by Xie et al. (2006), the CCPS consists of 23 items divided into two subscales. The cooperation subscale (13 items) covers reciprocity, acceptance and gregariousness; the competition subscale (10 items) includes self-growth, superiority pursuit and hyper-competitiveness. Higher scores indicate stronger corresponding personality tendencies. The reliability coefficients of the cooperation and competition subscales are 0.85 and 0.71, respectively, and confirmatory factor analysis supports satisfactory construct validity. In the present study, participants with extreme scores (top and bottom 27%) in either subscale were excluded to eliminate the influence of extreme personality traits and ensure balanced cooperative and competitive tendencies across groups.

④ Eysenck Personality Questionnaire-Adult (EPQ-A)

Developed by Eysenck (1975; Eysenck et al., 1991), this self-report scale has adult (88 items) and junior (81 items) versions, comprising four subscales: Extraversion (E), Neuroticism (N), Psychoticism (P), and Lie scale (L). With high reliability and validity, it is widely applied in personality research. Only the 21-item E subscale was used to assess extraversion-introversion traits, with test-retest reliability ranging from 0.80 to 0.96. Raw scores were converted into standardized T-scores via the formula:

T=50+10×(X−M)/SD

. Participants were classified as intermediate (43.3–56.7), inclined (38.5–43.3 or 56.7–61.5), or typical introversion/extraversion (<38.5 or >61.5). All participants were further divided into introverted and extraverted groups for pairing.

⑤ Intimacy Scale

This scale was used to measure participants’ intimate feelings before and after the experiment. A 7-point Likert scale was adopted to evaluate participants’ affection toward daily friends and unfamiliar experimental partners (Li et al., 2020), ranging from 1 (not at all) to 7 (extremely). Higher scores represent higher interpersonal intimacy.

⑥ Trust Scale

The scale assessed participants’ interpersonal trust before and after the experiment. With a 7-point Likert rating (1 = not at all, 7 = extremely), it measured trust in daily friends and experimental partners (Li et al., 2020). Higher scores indicate a higher level of interpersonal trust.

⑦ Subjective Cooperativeness Scale

This scale evaluated participants’ subjective cooperative experience before and after the experiment (Li et al., 2020). A 7-point Likert scale was used to rate perceived cooperation and mutual rapport between partners. Higher scores reflect better subjective cooperative performance.

⑧ Self-Rating Anxiety Scale (SAS)

The SAS was compiled by Zung (1971), containing 20 items rated on a 4-point scale. The raw score (X) was summed and converted to a standard score with the formula

Y=int(1.25X)

. Items 5, 9, 13, 17, and 19 were reverse-scored. Based on Chinese norms, the cut-off standard score is 50: 50–59 for mild anxiety, 60–69 for moderate anxiety, and ≥70 for severe anxiety. Higher scores indicate more severe anxiety symptoms. Participants with a standard score ≥ 51 were excluded to control for anxiety confounders.

⑨ Beck Depression Inventory-II (BDI-II)

Compiled by Beck (1996), the BDI-II includes 21 items with a 0–3 scoring system to assess depressive symptoms and severity (Steer et al., 2000). Total scores were categorized as: no depression (0–13), mild depression (14–19), moderate depression (20–28), and severe depression (29–63). Participants with depressive tendencies were excluded in this experiment.

⑩ Interaction Anxiousness Scale (IAS)

Developed by Leary (1983), the 15-item IAS measures subjective social anxiety with a 1–5 rating scale. Items 3, 6, 10 and 15 were reverse-scored. Total scores range from 15 to 75, with higher scores indicating greater social anxiety. Individuals with prominent social anxiety tendencies were excluded.

⑪ College Students’ Social Exclusion Questionnaire

Compiled by Wu et al. (2013), this 19-item scale (1–5 scoring) evaluates chronic social exclusion experiences among Chinese college students, with higher scores indicating more frequent exclusion. To ensure intergroup homogeneity, participants with a mean item score ≥ 3 were excluded, while those scoring 1–2 were retained for formal participation.

⑫ Profile of Mood States-Short Form (POMS-SF)

Originally developed by McNair (1984) and revised for Chinese undergraduates by Zhu (1995), the 40-item POMS-SF adopts a 0–4 scoring system and consists of seven subscales. The total mood score was calculated as: Total negative emotion scores − Total positive emotion scores + 100. Based on the average score of negative emotion subscales, only participants with a mean score of 0–1 were included, and those with a mean score ≥ 2 were excluded to guarantee baseline emotional consistency.

⑬ Need to Belong Scale (NTB)

Developed by Leary et al. (2013), the 10-item NTB uses a 7-point Likert scale to measure the intensity of belonging needs. Higher scores reflect a stronger desire for interpersonal acceptance and affiliation. The scale was administered pre- and post-experiment to detect changes in belonging motivation induced by social exclusion.

⑭ State Self-Esteem Scale (SSES)

Constructed by Heatherton and Polivy (1991), the SSES contains 6 items covering social, appearance, and performance self-esteem, with a 1–4 Likert rating. Higher scores indicate higher momentary self-esteem. The scale was measured before and after social exclusion to capture dynamic fluctuations in state self-esteem.
